# Perinatal choline supplementation prevents learning and memory deficits and reduces brain amyloid Aβ42 deposition in *App*^NL-G-F^ Alzheimer’s disease model mice

**DOI:** 10.1371/journal.pone.0297289

**Published:** 2024-02-05

**Authors:** Thomas A. Bellio, Jessenia Y. Laguna-Torres, Mary S. Campion, Jay Chou, Sheila Yee, Jan K. Blusztajn, Tiffany J. Mellott

**Affiliations:** Department of Pathology & Laboratory Medicine, Boston University Chobanian & Avedisian School of Medicine, Boston, Massachusetts, United States of America; Veneto Institute of Oncology Institute for Hospitalization and Care Scientific: Istituto Oncologico Veneto Istituto di Ricovero e Cura a Carattere Scientifico, ITALY

## Abstract

Alzheimer’s disease (AD) is characterized by cognitive and memory impairments and neuropathological abnormalities. AD has no cure, inadequate treatment options, and a limited understanding of possible prevention measures. Previous studies have demonstrated that AD model mice that received a diet high in the essential nutrient choline had reduced amyloidosis, cholinergic deficits, and gliosis, and increased neurogenesis. In this study, we investigated the lifelong effects of perinatal choline supplementation on behavior, cognitive function, and amyloidosis in *App*^NL-G-F^ AD model mice. Pregnant and lactating mice were given a diet containing either 1.1 g/kg (control) or 5 g/kg (supplemented) of choline chloride until weaning and subsequently, all offspring received the control diet throughout their life. At 3, 6, 9, and 12 months of age, animals were behaviorally tested in the Open Field Test, Elevated Plus Maze, Barnes Maze, and in a contextual fear conditioning paradigm. Immunohistochemical analysis of Aβ42 was also conducted on the brains of these mice. *App*^NL-G-F^ mice displayed hippocampal-dependent spatial learning deficits starting at 3-months-old that persisted until 12-months-old. These spatial learning deficits were fully prevented by perinatal choline supplementation at young ages (3 and 6 months) but not in older mice (12 months). *App*^NL-G-F^ mice also had impaired fearful learning and memory at 9- and 12-months-old that were diminished by choline supplementation. Perinatal choline supplementation reduced Aβ42 deposition in the amygdala, cortex, and hippocampus of *App*^NL-G-F^ mice. Together, these results demonstrate that perinatal choline supplementation is capable of preventing cognitive deficits and dampening amyloidosis in *App*^NL-G-F^ mice and suggest that ensuring adequate choline consumption during early life may be a valuable method to prevent or reduce AD dementia and neuropathology.

## Introduction

Alzheimer’s disease (AD) is a progressive neurodegenerative disease and the most common form of dementia. AD is characterized clinically by cognitive deficits and neuropathologically by the accumulation of amyloid-beta (Aβ) plaques, neurofibrillary tangles, and neuroinflammation. Although AD was first described by Dr. Alois Alzheimer in 1907, effective treatment options for the disease have remained elusive. For this reason, there has been an increased interest in possible prevention methods for this disease. Choline is an essential nutrient used in the synthesis of phosphatidylcholine, is a precursor for the neurotransmitter acetylcholine, and may serve as a methyl group donor for the synthesis of S-adenosylmethionine which can be used to methylate multiple substrates, including DNA and histones [1]. Although choline is recognized as an essential nutrient, only about 10% of Americans reach its daily recommended intake level [2]. One of the hallmarks of AD is the loss of the basal forebrain cholinergic neurons that are critical for learning and memory, and perinatal choline supplementation in mice and rats increases the size of these neurons and enhances their acetylcholine synthesis and release [3–7]. Numerous animal studies have shown that maternal choline supplementation has lifelong beneficial effects on cognition and brain development in their offspring [3, 4, 8, 9]. Perinatal choline supplementation in rats has also been shown to enhance hippocampal long-term potentiation, improve spatial learning and memory, and to modulate the expression of genes encoding proteins that are critical for long-term potentiation and are involved in memory processing [4, 5, 10–15]. Choline supplementation also reduces brain amyloid deposition in APP.PS1 AD model mice [10, 16, 17]. In contrast, maternal choline deficiency has negative effects on brain development, cognitive performance, and adult neurogenesis [18, 19] and low levels of dietary choline intake are associated with AD-neuropathological progression and increased risk for dementia and AD [20, 21]. Together, these data support the notion that adequate choline consumption may be a potential avenue for AD prevention or risk mitigation.

In 2014, Saito and colleagues developed the *App*^NL-G-F^ AD mouse model with the goal to overcome one of the major disadvantages of the transgenic AD mouse models which are characterized by the overexpression of the amyloid precursor protein (*APP*) controlled by an exogenous promoter. Among other drawbacks, this led to an overproduction of APP C-terminal fragment (βCTF-β/α), and APP intracellular domain that is not apparent in AD in humans [22]. *App*^NL-G-F^ mice do not overexpress APP and thus are thought to better represent some aspects of AD. The *App*^NL-G-F^ model accomplishes this by knocking in a humanized version of the Aβ amino acid sequence into *App* while also incorporating the Swedish (KM670/671NL), Artic (E693G), and Beyrether/Iberian (I716F) mutations that lead to familial AD, in order to accelerate brain amyloidosis. *App*^NL-G-F^ AD model mice start developing plaques and signs of neuroinflammation starting around two months of age and these pathological hallmarks progress in an age-dependent manner [22, 23]. Spatial and fearful memory impairments have been reported starting at 6 months of age in *App*^NL-G-F^ mice and continue to be present at least up until 18-months-old [22–25]. Measures of anxiety in *App*^NL-G-F^ mice have been inconsistent with some studies finding slight differences at early ages that are then flipped the other direction at later ages or no real difference in anxiety in *App*^NL-G-F^ mice compared to wildtype [24, 26, 27]. Furthermore, anxiety measures in *App*^NL-G-F^ mice seem to be dependent on type of test, age, and testing environment. Although several studies have evaluated AD mouse models behaviorally and pathologically across various ages, none have investigated the effects of perinatal choline supplementation across the lifespan within any AD mouse model [23–33]. Choline supplementation has been shown to improve behavioral deficits in the APP.PS1 AD mouse model at 11 months of age [17] but the effects of choline supplementation on amyloidosis and behavior in the *App*^NL-G-F^ mouse model, and any AD mouse model, have not been studied across life. In this study, we investigated the effects of both the *App*^NL-G-F^ genotype and of perinatal choline supplementation on anxiety and exploratory behavior, spatial and fearful learning and memory, and Aβ42 pathology in 3-, 6-, 9-, and 12-month-old wildtype and *App*^NL-G-F^ mice.

## Materials and methods

### Ethics statement

All procedures were conducted in accordance with the Animal Welfare Act (Animal Welfare Assurance Number A-3316-01) and to the principles of the National Institute of Health Guide for the Care and Use of Laboratory Animals (“The Guide”). All studies were approved by the Institutional Animal Care and Use Committee of Boston University.

### Animals and animal care

C57BL/6J (WT) mice were purchased from Charles River Laboratories (Worcester, MA, USA) and then bred in-house. Knock-in *App* KM670/671NL (Swedish), E693G (Artic), I716F (Iberian) (*App*^NL-G-F^) mice were obtained from RIKEN BioResource Center under a Material Transfer Agreement and then bred in-house. One week prior to mating, mice were placed on either a control AIN76A diet (#110098 Dyets Inc., Bethleham, PA) consisting of carbohydrates (66.00%), protein (20.30%), and fat (5.00%), [as sucrose (500g/kg), casein (200g/kg), cornstarch (150g/kg), cellulose (50g/kg), corn oil (50g/kg), mineral mix #200000 (35g/kg), vitamin mix #300050 (10g/kg), DL-methionine (3g/kg), and choline chloride (1.1g/kg)], or on a choline-supplemented AIN76A diet (#110184 Dyets Inc., Bethleham, PA), differing only by containing 5.0 g/kg of choline chloride, creating control diet and choline-supplemented groups. Homozygous *App*^NL-G-F^ and C57BL/6J females were crossed correspondingly with homozygous *App*^NL-G-F^ and C57BL/6J males. The pregnant dams continued to consume their specific prenatal diets through the birth of the litter and lactation until weaning of the pups on postnatal day 21 (S1 Fig). Subsequently, all offspring consumed the control diet and water *ad libitum* and were randomly divided into four experimental age groups: 3-, 6-, 9-, and 12-months of age. Animals were group housed by sex with a maximum of 5 mice per cage with controlled ambient temperature and humidity between 20-22ºC and 50–70%, respectively. Mice were housed on a reverse 12-hour light-dark cycle (11 pm-11 am lights on). Mice were euthanized after completion of behavioral testing at 3-, 6-, 9-, or 12-months of age (S1 Fig).

### Behavioral testing

All mice were acclimated to the testing room at least 30 minutes prior to testing. Before and after individual tests, surfaces and testing apparatuses were sanitized with 70% ethanol. Mice were weighed before the Open Field Test and after Contextual Fear Conditioning to test for any significant weight gain or reduction during the behavioral testing period. There were no differences in mouse weight due to genotype or perinatal diet, nor did behavioral testing significantly alter mouse weight. During testing, all mice from one cage were placed in individual holding cages where they remained until the end of the testing sessions. Holding cages were used during the experiment to prevent the emotional reactivity of a mouse that just completed the task from influencing the behavior of an untested mouse, among other potential artifacts. Animal testing was conducted in a cross-sectional manner so every animal was tested only at one age. All mice were behaviorally tested in the following order: Open Field Test, Elevated Plus Maze, Barnes Maze, and Contextual Fear Conditioning. All tests were performed such that animals were given at least two days of no testing between different tasks: Open Field; 2-day break; Elevated Plus Maze; 2-day break; Barnes Maze; 3-day break, Contextual Fear Conditioning. Number of animals per group are shown in S1 Table.

#### Open field test

The Open Field Test was conducted using a 45 x 60 x 45 cm arena made of plexiglass [34]. Each mouse was placed in the corner and released to explore the apparatus for 15 minutes. Light illumination was set to 140 lux and white noise was set to 55 dB. Ethovision XT 14 recording software (version 14.0.132.6, Noldus Information Technology, Leesburg, VA, USA) was used to track and quantify the animal’s total distance traveled, velocity, and time spent in central (25 x 40 cm) and peripheral zones (within 10 cm around the perimeter).

#### Elevated plus maze

Animals were tested for anxiety-like behaviors in the Elevated Plus Maze (EPM) [35]. The EPM consisted of two open arms (30 × 10 cm) and two closed arms (30 × 10 × 20 cm) extending from a central area (10 × 10 cm) and elevated 46 cm above the testing table. Testing was completed using 290 lux light illumination and 55 dB of white noise. Testing commenced by placing and releasing the animal in the center of the apparatus. The animal was allowed to explore the maze for 5 minutes. Times spent in the open arms, center, and closed arms and the number of entries into the open arms and closed arms were recorded. An entry into the open or closed arm was called when all four paws of the mouse were within a given region. Test parameters were scored by Ethovision XT 14 recording software (version 14.0.132.6, Noldus Information Technology, Leesburg, VA, USA).

#### Barnes maze

To examine spatial learning and memory function, the mice were tested using the Barnes Maze [36]. A 92 cm diameter gray platform elevated 46 cm above the testing table with 20 holes (5 cm diameter) equally spaced 2.5 cm from the perimeter was used. Testing was conducted in a well-lit (270 lux) room with salient extra maze cues and a white noise machine producing noise at 68 dB. A dark escape pod (10 x 10 x 8 cm) was attached underneath one of the holes and held at a constant position throughout the entirety of testing. The animals were tested in in three phases: habituation (Day 1), training (Days 2–4), and probe/memory recall (Day 5). The habituation phase occurred on the first day in which each animal had one trial of two minutes to explore the maze. If the animal entered the escape pod, the trial was stopped and the animal returned to their home cage. However, if the animal did not enter the escape hole by the end of the 2-minute period, the animal was gently guided into the escape pod and let sit in the escape pod for 30 seconds before being returned to its home cage. Twenty-four hours after the habituation phase, the animals began the training phase in which the animals completed three trials per day for three consecutive days. During the training phase, each trial was a maximum of 90 seconds. Like the habituation phase, during the training phase, if the animal entered the escape pod before 90 seconds elapsed, the trial was stopped and the animal returned to its cage. If the animal did not enter the escape pod before the end of the 90 seconds, the animal was gently guided into the escape pod before being returned to its cage. During the habituation and training phases, latency to reach the target hole, latency to escape, and number of errors were recorded. Primary latency was defined as the time to identify the target hole the first time by placing the head within the threshold of the target hole, as mice did not always enter the target hole upon first identifying it. Errors were defined as the number of incorrect head pokes that broke the threshold of a non-target hole before reaching the target hole. The probe/memory recall phase occurred 24 hours after the last training day and consisted of only one trial that lasted 30 seconds where the escape hole was closed with an opaque plug to test how well the animal remembered where the escape pod was placed in the previous days’ trials. At the end of the 30 seconds, the animal was returned to its home cage. Measures of latency to reach target hole, time spent per quadrant, mean distance from target hole, and number of errors were recorded for the probe/memory recall phase. For each trial, test parameters were scored by Ethovision XT 14 recording software (version 14.0.132.6, Noldus Information Technology, Leesburg, VA, USA).

#### Contextual fear conditioning

Contextual fear conditioning (CFC) was used to evaluate amygdala and hippocampal-dependent fearful learning and memory [37]. CFC was conducted in three sessions separated by 24 hours: a training session (Day 1) and two re-exposure sessions (Day 2 and Day 3). On Day 1, animals were placed in the conditioning chamber (17 x 17 x 25 cm) with white wall plates and a wire floor (Context 1) and allowed to habituate for 2 minutes before the onset of the first trial tone (20 seconds, 2000 Hz). A mild electric shock (2 seconds, 0.5 mA) was administered via the wire floor during the last 2 seconds of the tone followed by a resting period of 100 seconds. Each animal received five of these conditioning trials on Day 1 and remained in the conditioning chamber for 2 minutes after the last shock delivery before being returned to their cage. On Day 2, animals were placed in the same chamber but with black-and-white striped-wall plates and a solid floor (Context 2) to assess their response to the tone. After a 2-minute habituation, a tone (20 seconds, 2000 Hz) was administered in the absence of any shock and repeated for a total of five tones separated by 100 seconds each. On Day 3, animals were placed in the original context (Context 1) for 5 minutes without the presence of any tone or shock to assess context association. All testing was done using 240 lux illumination. The murine fear response in anticipation of the shock was measured using Ethovision XT 14 recording software (version 14.0.132.6, Noldus Information Technology, Leesburg, VA, USA) as percentage of time spent freezing (absence of all but respiratory movements for at least 3 seconds) to assess emotional reactivity during training (Day 1) and fear memory during retention tests (Day 2 and 3).

### Brain processing

Mice were humanely euthanized one week after completion of behavioral testing at 3-, 6-, 9-, or 12-months of age by CO_2_ inhalation followed by decapitation. Brains were quickly extracted and sectioned down the midline. The right hemisphere was immediately fixed in 10 volumes of PLP fixative (10mM sodium periodate, 75mM lysine, 4% paraformaldehyde; pH 7.4) for 24 hours at 4ºC, then cryoprotected in a graded series of 10% and 20% glycerol/2% dimethylsulfoxide, in 0.1 M phosphate buffer, pH 7.3 solution for 24 hours each. Serial, frozen sections (40μm, coronal) were cut from anterior to posterior on a frozen sliding microtome and cut sections stored in a 0.02% sodium azide in 0.1 M PBS (pH 7.3) solution until time of staining.

### Amyloid-beta-42 immunohistochemistry

Anterior hippocampal sections that capture the hippocampus, primary somatosensory cortex, and basolateral and basomedial amydalar nuclei (Bregma -1.5mm—-1.9mm) were selected and washed with gentle agitation for 10 minutes in PBS and then transferred to 70% Formic Acid for 1 minute with gentle agitation. The sections were blocked in 0.1 M PBS (pH 7.3)/10% goat serum for 1 hour at room temperature. Sections were incubated with rabbit anti-Aβ42 antibody (Invitrogen #700254; 1:1000) overnight with gentle agitation at room temperature in a solution of 0.3% Triton-X 100, 2% goat serum, 0.008% sodium azide, in 0.1M PBS (pH 7.3). The next day, sections were washed with 0.1 M PBS (pH 7.3) three times for 10 minutes each before being incubated with goat anti-rabbit IgG (H+L) HRP conjugated antibody (Millipore #AP307P; 1:750) in a solution of 2% goat serum/0.1 M PBS (pH 7.3) for 3 hours with gentle agitation at room temperature. Sections were then washed with 0.1 M PBS (pH 7.3) three times for 10 minutes each before being developed in a solution containing diaminobenzidine, sodium imidazole, and hydrogen peroxide. These immunohistochemical procedures on sections slated to constitute a set used for comparative studies were performed at the same time with the same reagents under identical conditions. Mounted sections were analyzed on an Olympus B061 microscope using a 2X magnification objective. The entire hippocampus and adjacent cerebral cortex were imaged in a single photographic frame, and a second set of images that contained the amygdala were imaged subsequently. The photographic images were obtained using constant exposure settings for each set of sections. Using the ImageJ software, the region of interest was outlined to include the entire hippocampus, a consistent region of the cerebral cortex, and a consistent region in the amygdala encompassing both the basolateral and basomedial amygdalar nuceli in each of the images (S2 Fig). Number of animals ranged from 5–11 per group (Age by perinatal diet by sex) and shown in S2 Table. The staining intensity threshold was held constant for all of the images in a given set. Total Aβ42-positive plaque number, average Aβ42-positive plaque size, and Aβ42-positive total plaque area were measured by the ImageJ software. Three sections per animal were used, and the data averaged to obtain a single value for the anterior hippocampus, cerebral cortex, and amygdala of that animal. Then, the data were used to calculate the mean and standard error for each group of animals per region. The analysis was performed by an individual (MC), who was blind to the identity of the samples (dietary group, age, and sex status).

### Data analysis

Data for all experiments, presented as means ± SEM, were analyzed by t-test or a one- or two- way ANOVA or ANOVA with repeated measures as appropriate using Jmp Pro 15.0.0 software. *Post hoc* analyses were performed with a Tukey test. Sex was included as a covariate in all analyses and sex-related differences were evaluated and only reported when significant. For figures p-values are designated with asterisks, *<0.05; **<0.01; ***<0.001.

## Results

### Open field test and elevated plus maze

We employed both the Open Field Test and Elevated Plus Maze to explore anxiety-related behaviors and locomotion within our mice. We found no differences between wildtype and *App*^NL-G-F^ mice in total distance traveled during the Open Field Test at any of the ages indicating that *App*^NL-G-F^ mice did not display locomotor abnormalities (p>0.05 for all, ANOVA, S3A Fig). *App*^NL-G-F^ mice did, however, show subtle anxiety-related differences compared to wildtype mice. In the Open Field Test, there was an overall significant effect of the *App*^NL-G-F^ genotype as *App*^NL-G-F^ mice spent less time in the center than wildtype mice at both 9- and 12-months of age (9-Months- F(1, 65) = 5.50, p = 0.022; 12-Months- F(1, 62) = 5.51, p = 0.022; ANOVA, S3B Fig). In the Elevated Plus Maze, there was also a significant effect of genotype, as *App*^NL-G-F^ mice spent significantly more time in the open arms or middle of the platform than wildtype mice, but only at 12-months of age (F(1, 62) = 6.91, p = 0.011; ANOVA, S4B Fig). No other genotype differences were found in the Open Field Test at 3- or 6-months of age or in the Elevated Plus Maze at 3-, 6-, or 9-months of age (S3 and S4 Figs). Together, these data show that *App*^NL-G-F^ mice do not have locomotor dysfunction and have subtle anxiety-related differences as compared to wildtype mice. These subtle changes found in the Open Field Test and Elevated Plus Maze are unlikely to explain the marked performance differences between *App*^NL-G-F^ and wildtype mice in learning and memory tests like the Barnes Maze and Contextual Fear Conditioning reported below. Further detailed analysis of both the Open Field Test and Elevated Plus Maze is included in the S1 File.

### Barnes maze

**Fig 1:**
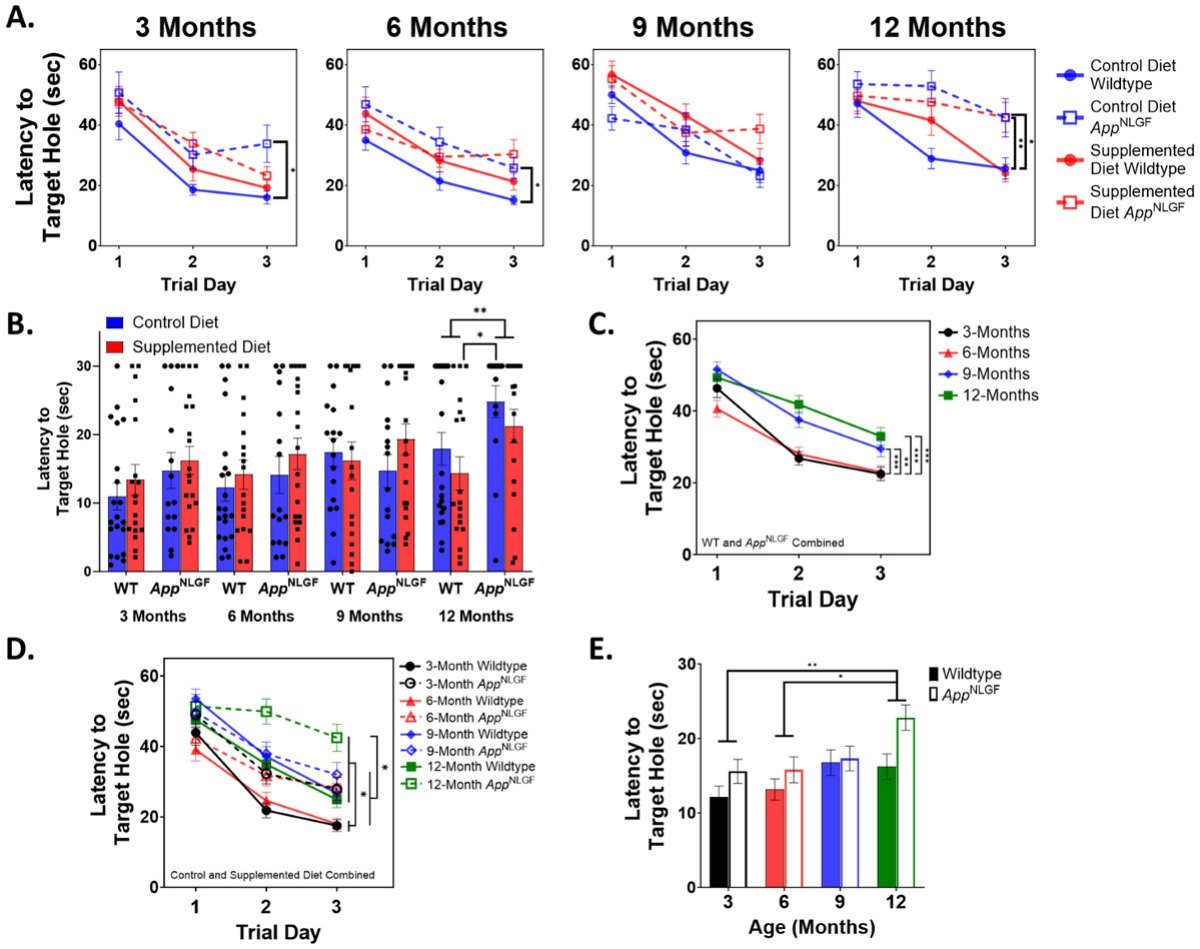
*App*^NL-G-F^ mice are impaired in spatial learning and perinatal choline supplementation can prevent these deficits at early ages. (A) *App*^NL-G-F^ mice at 3-, 6-, and 12-months of age take a longer time to reach the target hole than wildtype mice (3-Months- F(1, 64) = 9.99, p = 0.002; 6-Months- F(1, 67) = 5.75, p = 0.019; 12-Months- F(1, 62) = 14.40, p = 0.0003; repeated measures ANOVA). Perinatal choline supplementation prevented these learning deficits in *App*^NL-G-F^ mice at 3- and 6-months of age but not at 12-months. (B) No significant spatial memory recall deficits were found in 3-, 6-, or 9-month-old *App*^NL-G-F^ mice but there was a spatial memory recall deficit in *App*^NL-G-F^ mice at 12-months-old revealed during the 1-day probe test (F(1, 66) = 7.43, p = 0.008, ANOVA). 12-month-old *App*^NL-G-F^ mice that received the control diet were significantly slower at reaching the target hole during the 1-day probe test than 12-month-old wildtype mice that received the supplemented diet (Supplemented WT (14.32 ± 2.4s) vs Control *App*^NL-G-F^ (24.79 ± 2.7s) p = 0.025, Tukey). (C) Linear regression models adjusting for sex, perinatal diet, and genotype of latency to target hole measures during the trial days of the Barnes Maze revealed that 3- and 6-month-old mice are significantly faster at reaching the target hole than both 9- and 12-month-old mice (3-Months (31.96 ± 1.4s) vs 9-Months (39.10 ± 1.4s) p = 0.003; 3-Months (31.96 ± 1.4s) vs 12-Months (41.41 ± 1.5s) p<0.0001; 6-Months (30.94 ± 1.4s) vs 9-Months (39.10 ± 1.4s) p = 0.0004; 6-Months (30.94 ± 1.4s) vs 12-Months (41.41 ± 1.5s) p<0.0001; Tukey). Wildtype and *App*^NL-G-F^ were combined for these analyses. (D) Linear regression models adjusting for trial day, sex, and perinatal diet of latency to target hole during the trial days of the Barnes Maze show that young (3- and 6-month) *App*^NL-G-F^ mice perform more like older (9- and 12-month) wildtype mice than their age-matched counterparts. Significance bars shown indicate differences between 12-month-old *App*^NL-G-F^ mice and all other groups (p<0.03 for all, Tukey), as well as the differences between 3- and 6-month-old wildtype mice and all other groups (p<0.05 for all, Tukey). (E) In the 1-day probe test, 3- and 6-month-old mice perform significantly better than 12-month-old mice (3-Months (13.92 ± 1.1s) vs 12-Months (19.06 ± 1.1s) p = 0.007; 6-Months (14.44 ± 1.1s) vs 12-Months (19.06 ± 1.1s) p = 0.019; Tukey).

To assess hippocampal-dependent spatial learning and memory, we performed the Barnes Maze that included a habituation day followed by three trial days with three tests per day and concluded with a probe test 24-hours after the last test of the last trial day. In 3-month-old mice, both wildtype and *App*^NL-G-F^ mice decreased their latency to the target hole across the trial days indicating that the animals were learning the location of the hole (Wildtype- F(2, 102) = 28.75, p<0.0001; *App*^NL-G-F^- F(2, 90) = 11.72, p<0.0001; Fig 1A). There was an overall significant effect of genotype on latency to reach the target hole during the trial days as 3-month-old *App*^NL-G-F^ mice took significantly longer to reach the target hole than wildtype mice (F(1, 64) = 9.99, p = 0.002, repeated measures ANOVA). There was also an overall effect of genotype by perinatal diet during the trial days in 3-month-old mice (F(3, 64) = 3.97, p = 0.012, repeated measures ANOVA). *Post-hoc* analysis found that the genotype by perinatal diet effect was due to the significant increase in latency to reach the target hole during the trial days in control diet *App*^NL-G-F^ mice (38.67 ± 3.3s) compared to control diet wildtype mice (24.95 ± 2.8s) (p = 0.012, Tukey). However, 3-month-old *App*^NL-G-F^ mice that received the choline supplemented perinatal diet did not show a significant impairment in learning the location of the target hole compared to both control diet and choline supplemented wildtype mice (Control WT (24.95 ± 2.8s), Supplemented WT (29.45 ± 3.0s), Supplemented *App*^NL-G-F^ (34.79 ± 2.9s); Control WT vs Supplemented *App*^NL-G-F^ p = 0.0820; Supplemented WT vs Supplemented *App*^NL-G-F^ p = 0.585, Tukey, Fig 1A). This suggests that perinatal choline supplementation prevented the spatial learning deficits in 3-month-old *App*^NL-G-F^ mice (Fig 1A). This learning deficit during the trial days of the Barnes Maze in 3-month-old *App*^NL-G-F^ mice did not translate to a large enough effect in recall during the 1-day probe test as, although *App*^NL-G-F^ mice took longer to reach the target hole than wildtype mice, there was not a significant effect of genotype (F(1, 64) = 2.26, p = 0.137; ANOVA, Fig 1B) nor a significant effect of genotype by perinatal diet (F(3, 62) = 1.11, p = 0.352; ANOVA, Fig 1B). There was no significant overall effect of sex or perinatal diet on latency to the target hole in the 1-day probe test of 3-month-old mice (Fig 1 and S5B Fig).

Similarly, both wildtype and *App*^NL-G-F^ 6-month-old mice learned the target hole location during the trial day phase (WT- F(2, 74) = 25.90, p<0.0001; *App*^NL-G-F^- F(2, 68) = 8.07, p = 0.0007; repeated measures ANOVA, Fig 1A). There was a significant overall effect of genotype as 6-month-old *App*^NL-G-F^ mice took a significantly longer time to find the target hole during the trial days than 6-month-old wildtype mice (F(1, 67) = 5.75, p = 0.019; repeated measures ANOVA, Fig 1A). Again, there was a significant effect of genotype by perinatal diet (F(3, 71) = 3.36, p = 0.024, repeated measures ANOVA). Just like in 3-month-old mice, the genotype by perinatal diet effect was driven by the deficit in latency to the target hole during the trial days in control diet 6-month-old *App*^NL-G-F^ mice compared to control diet 6-month-old wildtype mice (Control WT (23.86 ± 2.6s) vs Control *App*^NL-G-F^ (35.69 ± 3.0s) (p = 0.022, Tukey). Remarkably, 6-month-old *App*^NL-G-F^ mice that had received the choline supplemented diet did not show this spatial learning deficit during the trial days compared to both control and supplemented wildtype mice (Control WT (23.86 ± 2.6s) vs Supplemented *App*^NL-G-F^ (32.79 ± 2.7s) p = 0.093; Supplemented WT (31.11 ± 2.8s) vs Supplemented *App*^NL-G-F^ (32.79 ± 2.7s) p = 0.974; Tukey, Fig 1A). Further matching the 3-month-old results, although there was a trend for 6-month-old *App*^NL-G-F^ mice to take a longer time to reach the target hole than wildtype mice in the 1-day probe test, there was not a statistically significant effect of genotype (F(1, 73) = 1.20, p = 0.278; ANOVA) nor was there a significant genotype by perinatal diet effect (F(3, 71) = 0.87, p = 0.459; ANOVA, Fig 1B). There was also no effect of perinatal choline supplementation on latency to the target hole during the 1-day probe test in 6-month-old mice (Fig 1B). There was a significant sex difference in latency to the target hole in the 1-day probe test as 6-month-old female mice found the target hole faster than 6-month-old male mice (F(1, 73) = 7.92, p = 0.006; ANOVA, S5B Fig).

In both 9-month-old wildtype and *App*^NL-G-F^ mice, latency to reach the target hole decreased across the three-day trial phase (WT- F(2, 69) = 29.47, p<0.0001; *App*^NL-G-F^- F(2, 72) = 9.34, p = 0.0002, repeated measures ANOVA). Surprisingly, there was no overall effect of genotype in 9-month-old mice on latency to the target hole during the three-day trial phase like there was in 3- and 6-month-old mice as *App*^NL-G-F^ mice did not find the target hole at a rate that differed from wildtype mice (F(1, 64) = 0.009, p = 0.923, repeated measures ANOVA, Fig 1A). There was however, a sex difference as 9-month-old female mice were significantly faster at reaching the target hole compared to male mice across the three-day trial phase (F(1, 64) = 14.81, p = 0.0003, repeated measures ANOVA, S5A Fig). These trial day results were matched in the 1-day probe test as there was no significant effect of genotype in 9-month-old mice on latency to reach the target hole (F(1, 64) = 0.0005, p = 0.983, ANOVA, Fig 1B) but there was a sex difference as female mice made it to the target hole drastically faster than their male counterparts in the 1-day probe test (F(1, 64) = 15.89, p = 0.0002, ANOVA, S5B Fig).

At 12 months of age, wildtype mice decreased their latency to the target hole across the trial days (F(2, 74) = 17.64, p<0.0001; repeated measures ANOVA, Fig 1A); while, *App*^NL-G-F^ mice did not (F(2, 62) = 2.46, p = 0.094; repeated measures ANOVA, Fig 1A). This learning deficit across the trial days of the Barnes Maze in *App*^NL-G-F^ mice was true in both *App*^NL-G-F^ mice that received the control diet and those that received the choline supplemented diet perinatally (Control *App*^NL-G-F^- F(2, 26) = 2.14, p = 0.137; Supplemented *App*^NL-G-F^- F(2, 34) = 0.71, p = 0.498; repeated measures ANOVA, Fig 1A). This led to a significant effect of genotype as 12-month-old *App*^NL-G-F^ mice took a significantly longer time on average to reach the target hole than 12-month-old wildtype mice during the three trial days (F(1, 62) = 14.40, p = 0.0003; repeated measures ANOVA). There was a significant effect of genotype by perinatal diet in 12-month-old mice on latency to the target hole during the trial phase of the Barnes Maze (F(3, 62) = 5.14, p = 0.003; repeated measures ANOVA, Fig 1A). *Post-hoc* analysis revealed that the genotype by perinatal diet effect was driven by the learning deficit found in control diet *App*^NL-G-F^ mice compared to control diet wildtype mice (Control WT (33.87 ± 2.9s) vs Control *App*^NL-G-F^ (49.61 ± 3.5s) p = 0.005; Tukey, Fig 1A). However, unlike at 3 and 6 months of age, perinatal choline supplementation in 12-month-old *App*^NL-G-F^ mice was unable to fully prevent these spatial learning deficits as they performed significantly worse during the trial phase of the Barnes Maze than control diet wildtype animals (Control WT (33.87 ± 2.9s) vs Supplemented *App*^NL-G-F^ (46.58 ± 3.1s) p = 0.0182; Tukey, Fig 1A). Unlike mice that were 3, 6, or 9 months of age, there was a significant effect of genotype in the 1-day probe test as 12-month-old *App*^NL-G-F^ mice showed a significant impairment in spatial memory recall as they took significantly longer to reach the target hole than wildtype mice (F(1, 66) = 7.43, p = 0.008; ANOVA, Fig 1B). There was a significant effect of genotype by perinatal diet on latency to the target hole in the 1-day probe test in 12-month-old mice (F(3, 66) = 3.18, p = 0.030; ANOVA, Fig 1B). *Post-hoc* analysis revealed that the genotype by perinatal diet effect was driven by the significant difference between supplemented wildtype and control *App*^NL-G-F^ mice (Supplemented WT (14.32 ± 2.4s) vs Control *App*^NL-G-F^ (24.79 ± 2.7s) p = 0.025; Tukey, Fig 1B). There was also a significant sex difference in 12-month-old mice in the 1-day probe test as female mice reached the target hole faster than male mice (F(1, 66) = 6.86, p = 0.011; ANOVA, S5B Fig). These results show that *App*^NL-G-F^ mice are significantly impaired at hippocampal-dependent spatial learning compared to wildtype mice and that choline supplementation can prevent these deficits at early ages (3 and 6 months) but not at a later age (12 months).

We then used a linear regression model adjusting for trial day, sex, genotype, and perinatal diet to evaluate the effects of age on latency to reach the target hole during the trial days and found that age significantly affected this measure during the trial days of the Barnes Maze (F(3, 257.6) = 12.98, p<0.0001, MANOVA). *Post-hoc* analysis revealed that, when adjusting for trial day, sex, diet, and genotype, 3-month-old mice were significantly faster at reaching the target hole during the trial days than both 9- and 12-month-old mice (3-Months (31.96 ± 1.4s) vs 9-Months (39.10 ± 1.4s) p = 0.003; 3-Months (31.96 ± 1.4s) vs 12-Months (41.41 ± 1.5s) p<0.0001; Tukey, Fig 1C). Six-month-old mice were also significantly faster than 9- and 12-month-old mice (6-Months (30.94 ± 1.4s) vs 9-Months (39.10 ± 1.4s) p = 0.0004; 6-Months (30.94 ± 1.4s) vs 12-Months (41.41 ± 1.5s) p<0.0001; Tukey, Fig 1C) but no different than 3-month-old mice (3-Months (31.96 ± 1.4s) vs 6-Months (30.94 ± 1.4s) p = 0.957; Tukey, Fig 1C). Nine- and 12-month-old mice performed about equally as poorly as each other during the trial days (9-Months (39.10 ± 1.4s) vs 12-Months (41.41 ± 1.5s) p = 0.671; Tukey, Fig 1C). Additional *post-hoc* analyses using this linear regression model to investigate genotype by age effects revealed that *App*^NL-G-F^ mice more closely resemble older wildtype mice than their age-matched counterparts. For example, 12-month-old wildtype mice perform about equally as well as 3-month-old *App*^NL-G-F^ mice during the trial days of the Barnes Maze (3-Month *App*^NL-G-F^ (36.73 ± 2.1s) vs 12-Month WT (35.60 ± 2.0) p = 0.999, Tukey, Fig 1D). Furthermore, 12-month-old *App*^NL-G-F^ mice performed significantly worse than all other age by genotype groups (p<0.03 for all, Tukey, Fig 1D). During the trial phase of the Barnes Maze, 9- and 12-month-old wildtype mice found the target hole at about the same speed as 3-, 6-, and 9-month-old *App*^NL-G-F^ mice (p>0.4464 for all comparisons, Tukey, Fig 1D). And 3- and 6-month-old wildtype mice found the target hole during the trial days significantly faster than all other age by genotype groups (p<0.05 for all comparisons, Tukey, Fig 1D).

We then used a linear regression model adjusting for sex, genotype, and perinatal diet to investigate the effects of age on latency to reach the target hole during the 1-day probe test and found that age also greatly affects this measure (F(3, 272) = 12.53, p = 0.0005; ANOVA). When adjusting for sex, perinatal diet, and genotype, both 3- and 6-month-old mice were significantly faster at reaching the target hole during the 1-day probe test than 12-month-old mice (3-Months (13.92 ± 1.1s) vs 12-Months (19.06 ± 1.1s) p = 0.007; 6-Months (14.44 ± 1.1s) vs 12-Months (19.06 ± 1.1s) p = 0.019; Tukey, Fig 1E). Although, the latency steadily increased with age from 3 to 9 months, there was no statistical difference between 3-, 6-, and 9-month-old mice in the 1-day probe test. Much like the trial day results, the 1-day probe test also revealed that younger *App*^NL-G-F^ mice perform more similarly to older wildtype mice than their age-matched counterparts (Fig 1E). For example, 12-month-old wildtype mice performed about as equally well as 3-month-old *App*^NL-G-F^ mice in the 1-day probe test as there was only about a quarter of a second difference in finding the target hole between the two (3-Month *App*^NL-G-F^ (15.60 ± 1.6s) vs 12-Month WT (15.86 ± 1.5s) p = 0.999; Tukey, Fig 1E). Together, these results support the notion that *App*^NL-G-F^ mice have significant hippocampal-dependent spatial learning and memory deficits and, even at a young age, are more closely performing at the level of much older wildtype mice.

### Contextual fear conditioning

**Fig 2:**
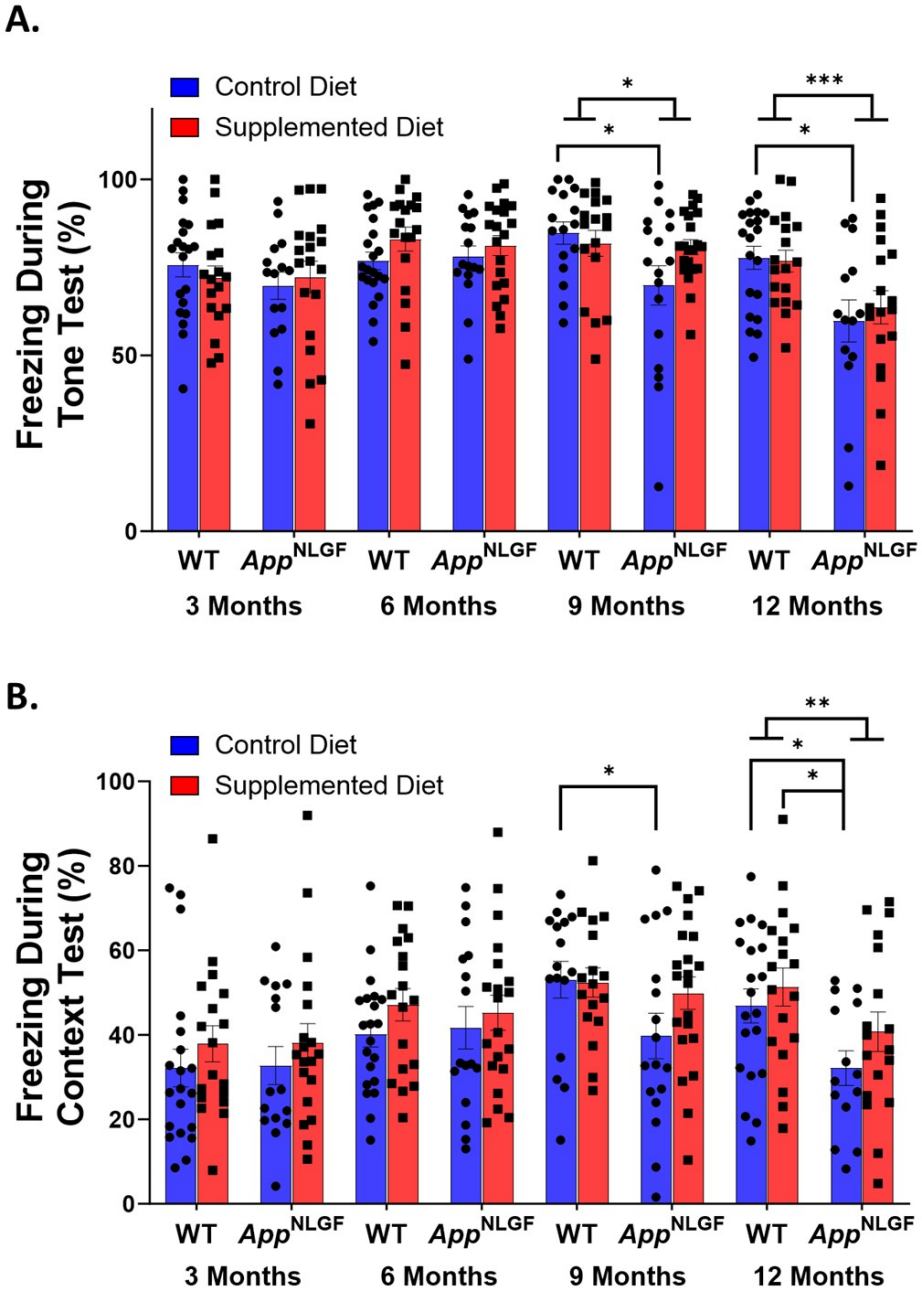
*App*^NL-G-F^ mice display impaired fearful learning and memory at 9- and 12-months-old that can be somewhat prevented by perinatal choline supplementation. (A) Percent of time freezing during the tone test of contextual fear conditioning revealed significant effect of genotype as *App*^NL-G-F^ mice froze significantly less than wildtype mice at 9- and 12-months of age (9-Months- F(1, 63) = 4.33, p = 0.042; 12-Months- F(1, 62) = 12.06, p = 0.0009; ANOVA). This deficit could be prevented by perinatal choline supplementation at 9- and 12-months of age. (B) Percent of time freezing during the context test of contextual fear conditioning revealed that control diet *App*^NL-G-F^ mice froze significantly less than control diet wildtype mice at 9- and 12-months of age and that perinatal choline supplementation could prevent these deficits at both ages (9-Months- Control WT (52.99 ± 4.7%) vs Control *App*^NL-G-F^ (39.69 ± 4.5%) p = 0.032; 12-Months- Control WT (46.84 ± 3.7%) vs Control *App*^NL-G-F^ (32.27 ± 4.6%) p = 0.016; Tukey). There was a significant effect of genotype in 12-month-old mice in the context test as 12-month-old *App*^NL-G-F^ mice spend significantly less time freezing during the context test than wildtype mice (F(1, 62) = 9.74, p = 0.003; ANOVA).

Contextual fear conditioning is a commonly used Pavlovian conditioning paradigm. Here, we used a three-day design that involved a training session on the first day where the mice were exposed to a loud tone followed by a foot shock that was repeated five times. On the second day, the tone test was completed in which the mice were placed into the conditioning chamber with a different floor and colored walls than the first day and the tone was played five times. On the third and final day, the context test was completed where the mice were placed into the conditioning chamber with the exact same floor and walls as the first day of the paradigm. Freezing—a total lack of movement for 3 seconds—was measured each day as a proxy to evaluate fearful learning and memory of both the tone and context of the fearful event. All mice associated the tone and the context with the shock as demonstrated by freezing behavior during a significant proportion of the time in the apparatus (Fig 2A and 2B).

In 3-month-old mice there was no significant effect of genotype as there was no difference between wildtype and *App*^NL-G-F^ mice on percent of time spent freezing during the tone test (F(1, 64) = 0.50, p = 0.482; ANOVA, Fig 2A) nor was there a genotype by perinatal diet effect on percent of time spent freezing during the tone test at this age (F(3, 64) = 0.52, p = 0.667; ANOVA, Fig 2A). There was, however, a significant effect of sex as three-month-old male mice spent a significantly larger percentage of time during the tone test freezing than 3-month-old female mice leading to a significant effect of sex on percent of time spent freezing during the tone test (F(1, 64) = 4.87, p = 0.031; ANOVA, S6A Fig). In the context test, there was also no significant effect of genotype (F(1, 64) = 0.04, p = 0.834; ANOVA, Fig 2B), genotype by perinatal diet (F(3, 64) = 0.37, p = 0.777; ANOVA, Fig 2B), or sex (F(1, 64) = 2.14, p = 0.148; ANOVA, S6B Fig) on percent of time spent freezing in 3-month-old mice.

In 6-month-old mice, there was no significant effect of genotype in both the tone test and the context test of contextual fear conditioning indicating that there is not an impairment of fearful learning and memory in *App*^NL-G-F^ mice at early ages (Tone Test- F(1, 67) = 0.009, p = 0.923; Context Test- F(1, 67) = 0.022, p = 0.884; ANOVA, Fig 2A and 2B). There was also no effect of genotype by perinatal diet in 6-month-old mice on both percent of time freezing during the tone test and during the context test (Tone Test- F(3, 67) = 1.03, p = 0.385; Context Test- F(3, 67) = 0.40, p = 0.753; ANOVA, Fig 2A and 2B).

There was a significant overall effect of genotype on percent of time freezing during the tone test in 9-month-old mice as *App*^NL-G-F^ mice spent significantly less time freezing than wildtype mice suggesting a deficit in fearful learning and memory at this age (F(1, 63) = 4.33, p = 0.042; ANOVA, Fig 2A). There was also a significant effect of genotype by perinatal diet on percent of time freezing during the tone test (F(3, 63) = 2.75, p = 0.0498; ANOVA, Fig 2A). *Post-hoc* analysis revealed that this genotype by perinatal diet effect was due to the impaired ability of control diet *App*^NL-G-F^ mice to freeze during the tone test compared to control diet wildtype mice (Control WT (85.09 ± 4.0%) vs Control *App*^NL-G-F^ (70.14 ± 3.9%) p = 0.045; Tukey, Fig 2A). Importantly, 9-month-old *App*^NL-G-F^ mice that had received the choline supplemented diet perinatally did not show any significant fearful learning and memory impairments during the tone test as there was minimal difference in performance between supplemented *App*^NL-G-F^ mice and both control wildtype and supplemented wildtype mice (Control WT (85.09 ± 4.0%) vs Supplemented *App*^NL-G-F^ (80.79 ± 3.5%) p = 0.849; Supplemented WT (81.68 ± 3.9%) vs Supplemented *App*^NL-G-F^ (80.79 ± 3.5%) p = 0.998; Tukey, Fig 2A). This suggests that perinatal choline supplementation ameliorates the fearful learning and memory deficits found in the tone test in 9-month-old *App*^NL-G-F^ mice on a control diet. These results were matched in the context test as 9-month-old *App*^NL-G-F^ mice that received the control diet froze significantly less than wildtype mice who also received the control diet (Control WT (52.99 ± 4.7%) vs Control *App*^NL-G-F^ (39.70 ± 4.5%) p = 0.032; Tukey, Fig 2B). Again, *App*^NL-G-F^ mice that had received the choline supplemented diet did not show a significant fearful learning and memory impairment as their performance in the context test was at about the same level as both control wildtype and supplemented wildtype mice (Control WT (52.99 ± 4.7%) vs Supplemented *App*^NL-G-F^ (49.76 ± 4.1%) p = 0.954; Supplemented WT (52.45 ± 4.5%) vs Supplemented *App*^NL-G-F^ (49.76 ± 4.1%) p = 0.970; Tukey, Fig 2B).

At 12 months of age, there was a significant overall effect of genotype as *App*^NL-G-F^ mice spent a smaller percent of their time freezing during the tone test of contextual fear conditioning than wildtype mice suggesting that the *App*^NL-G-F^ mice did not remember the tone as well as wildtype mice (F(1, 62) = 12.06, p = 0.0009; ANOVA, Fig 2A). There was also a significant effect of genotype by perinatal diet on percent freezing during the tone test in 12-month-old mice (F(3, 62) = 4.06, p = 0.011; ANOVA). The genotype by perinatal diet effect was again driven by the impairment in control *App*^NL-G-F^ mice compared to control wildtype mice (Control WT (77.74 ± 4.0%) vs Control *App*^NL-G-F^ (60.16 ± 5.0%) p = 0.040; Tukey, Fig 2A). Although the percent of time freezing during the tone test was decreased in *App*^*NL-G-F*^ mice that received the choline supplemented diet perinatally, the difference was not significant enough to lead to significant difference between supplemented *App*^NL-G-F^ and both control wildtype and supplemented wildtype mice (Control WT (77.74 ± 4.0%) vs Supplemented *App*^NL-G-F^ (63.68 ± 4.2%) p = 0.087; Supplemented WT (76.70 ± 4.3%) vs Supplemented *App*^NL-G-F^ (63.68 ± 4.2%) p = 0.145; Tukey, Fig 2A). In the context test, there was also a significant overall effect of genotype as 12-month-old *App*^NL-G-F^ mice spent a significantly lower percent of their time freezing than wildtype mice again suggesting that the *App*^NL-G-F^ mice are impaired in fearful learning and memory (F(1, 62) = 9.74, p = 0.003; ANOVA, Fig 2B). Again there was an effect of genotype by perinatal diet on percent of time spent freezing during the context test (F3, 62) = 3.77, p = 0.015; ANOVA). *Post-hoc* analysis revealed that this genotype by perinatal diet effect was due to the impairment in control *App*^NL-G-F^ mice as they froze significantly less than both control wildtype and supplemented wildtype mice during the context test (Control WT (46.84 ± 3.7%) vs Control *App*^NL-G-F^ (32.27 ± 4.6%) p = 0.016; Supplemented WT (51.38 ± 3.9%) vs Control *App*^NL-G-F^ (32.27 ± 4.6%) p = 0.013; Tukey, Fig 2B). Perinatal choline supplementation within 12-month-old *App*^NL-G-F^ mice prevented these deficits in the context test as there was no difference in percent of time freezing between them and both control wildtype and supplemented wildtype mice (Control WT (46.84 ± 3.7%) vs Supplemented *App*^NL-G-F^ (40.74 ± 3.9%) p = 0.668; Supplemented WT (51.38 ± 3.9%) vs Supplemented *App*^NL-G-F^ (40.74 ± 3.9%) p = 0.227; Tukey, Fig 2B). There was also a significant overall effect of sex in 12-month-old mice as male mice spent significantly more time freezing than female mice during the context test (F(1, 62) = 7.15, p = 0.0096; ANOVA, S6B Fig). Together, these results demonstrate that *App*^NL-G-F^ have impaired fearful learning and memory starting by 9-months of age and that perinatal choline supplementation mitigates some of these deficits.

### Amyloid-beta-42 immunohistochemistry

**Fig 3:**
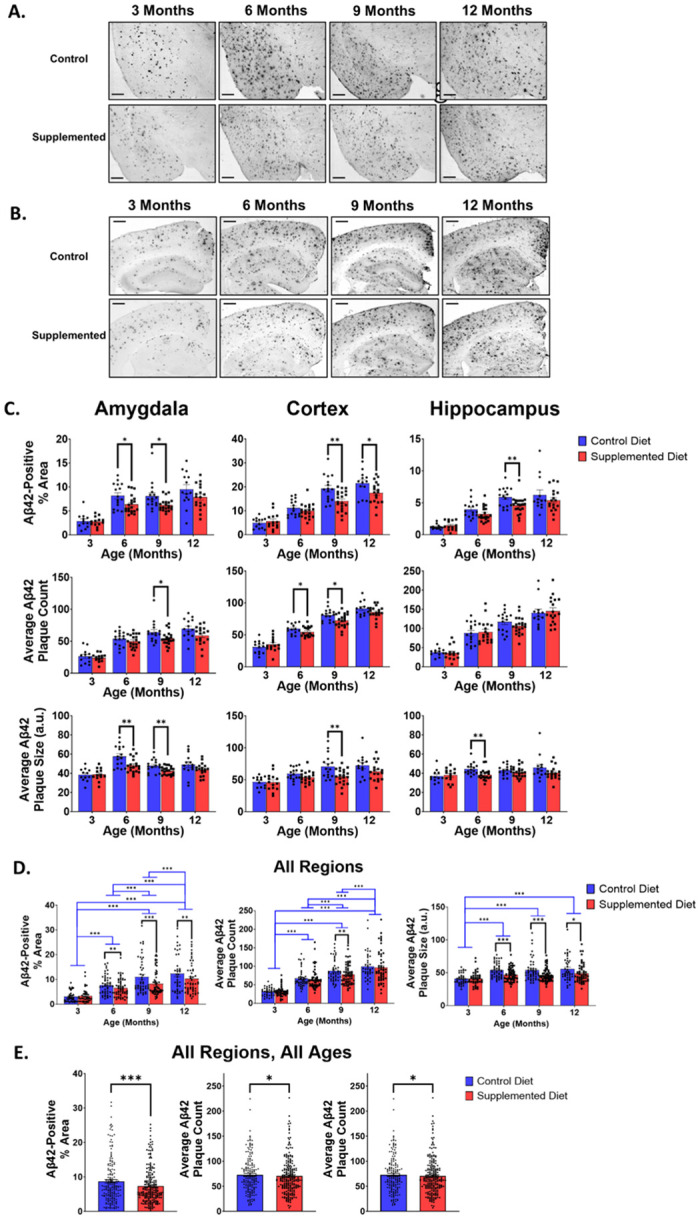
Perinatal choline supplementation reduces Aβ42 deposition in the amygdala, cortex, and hippocampus of *App*^NL-G-F^ mice. Representative 2x magnification images of the basolateral and basomedial amygdalar nuclei (A), hippocampus, and cortex (B) of Aβ42 immunohistochemistry in 3-, 6-, 9-, and 12-month-old mice show that Aβ42 deposits in an age-dependent manner and perinatal choline supplementation can reduce amyloidosis in these brain regions of *App*^NL-G-F^ mice. Scale bar is 400 μm. (C) Perinatal choline supplementation decreased Aβ42-positive percent area in the amygdala at 6- and 9-months of age, in the cortex at 9- and 12-months of age, and the hippocampus at 9-months of age (p = 0.022, 0.013, 0.0030, 0.042, 0.0099, respectively; ANOVA). Perinatal choline supplementation also reduced average Aβ42 plaque count in the amygdala at 9-months of age, and in the cortex at 6- and 9-months of age (p = 0.035, 0.046, 0.019, respectively; ANOVA) and reduced average Aβ42 plaque size in the amygdala at 6- and 9-months of age, in the cortex at 9-months of age, and in the hippocampus at 6-months of age (p = 0.005, 0.009, 0.004, 0.003, respectively; ANOVA). (D) Using linear regression modeling, perinatal choline supplementation was able to significantly reduce Aβ42-positive percent area at 6-, 9-, and 12-months of age (p = 0.006, 0.00002, 0.009, respectively; ANOVA, black bars), average Aβ42 plaque count at 9-months (p = 0.005; ANOVA, black bars), and average Aβ42 plaque size at 6-, 9, and 12-months of age (p = 0.00003, 0.0001, 0.018, respectively; ANOVA, black bars). 3-month-old mice had a significantly lower Aβ42-positive percent area, lower average Aβ42 plaque count, and smaller average Aβ42 plaque size than 6-, 9-, and 12-month-old mice (blue bars). 6-month-old mice had a significantly lower Aβ42-positive percent area and lower average Aβ42 plaque count than 9- and 12-month-old mice (blue bars). 9-month-old mice had a significantly lower average Aβ42 plaque count than 12-month-old mice. See *Results* for statistics. (E) Using linear regression models of Aβ42 immunohistochemistry across the amygdala, cortex, and hippocampus and all ages, perinatal choline supplementation was able to significantly reduce Aβ42-positive percent area, average Aβ42 plaque count, and average Aβ42 plaque size (p<0.0001, p = 0.047, p<0.0001, respectively; repeated measures ANOVA).

We and others have shown that both perinatal and lifelong choline supplementation can reduce amyloid pathology in the *APP*-overexpressing APP.PS1 mouse model of AD [10, 16, 38]. Here, we analyzed if perinatal choline supplementation would reduce Aβ42 pathology in the hippocampus, cortex, and amygdala across 3-, 6-, 9-, and 12-month ages in *App*^NL-G-F^ mice where *App* is expressed under the endogenous mouse promoter for the gene. We analyzed Aβ42 plaques using immunohistochemical staining quantified as: Aβ42-positive percent area, average Aβ42-positive plaque count, and average Aβ42-positive plaque size. As in previous studies, the *App*^NL-G-F^ mice were characterized by marked brain amyloidosis which increased with age [22, 23]. At 3 months of age, there was no significant difference between *App*^NL-G-F^ animals that received the control diet or the choline supplemented diet in Aβ42-positive percent area, average Aβ42-positive plaque count, or average Aβ42-positive plaque size in the hippocampus, cortex, or amygdala (Amygdala [% Area- F(1, 21) = 0.08, p = 0.781; Count- F(1, 21) = 0.34, p = 0.565; Size- F(1, 21) = 0.004, p = 0.949]; Cortex [% Area- F(1, 23)- 0.40, p = 0.533; Count- F(1, 23) = 0.34, p = 0.565; Size- F(1, 23) = 0.004, p = 0.949]; Hippocampus [% Area- F(1, 22) = 0.46, p = 0.505; Count- F(1, 22) = 0.45, p = 0.509; Size- F(1, 22) = 0.05, p = 0.82], ANOVA, Fig 3A–3C).

In 6-month-old *App*^NL-G-F^ mice, perinatal choline supplementation significantly reduced Aβ42-positive percent area and average Aβ42-positive plaque size in the amygdala compared to 6-month-old control diet *App*^NL-G-F^ mice (% Area- F(1, 32) = 5.77, p = 0.022; Size- F(1, 32) = 9.26, p = 0.005; ANOVA, Fig 3A and 3C). Although perinatal choline supplementation reduced the Aβ42-positive percent area and average Aβ42-positive plaque size in the amygdala, choline supplementation did not significantly reduce the Aβ42-positive plaque count in the amygdala of 6-month-old *App*^NL-G-F^ mice (F(1, 32) = 1.19, p = 0.028; ANOVA, Fig 3A and 3C). In the cortex of 6-month-old *App*^NL-G-F^ mice, perinatal choline supplementation reduced the average Aβ42-positive plaque count (F(1, 31) = 4.35, p = 0.046; ANOVA, Fig 3B and 3C). However, unlike in the amygdala, perinatal choline supplementation failed to reduce Aβ42-positive percent area and average Aβ42-positive plaque size in the cortex (% Area- F(1, 31) = 1.46, p = 0.236; Size- F(1, 31) = 3.13, p = 0.087; ANOVA, Fig 3B and 3C). Perinatal choline supplementation reduced the average Aβ42-positive plaque size in the hippocampus of 6-month-old *App*^NL-G-F^ mice that received the supplemented diet compared to 6-month-old *App*^NL-G-F^ mice raised on the control diet (F(1, 31) = 10.22, p = 0.003; ANOVA, Fig 3B and 3C). However, perinatal choline supplementation did not reduce the Aβ42-positive percent area or average Aβ42-positive plaque count in the hippocampus of 6-month-old *App*^NL-G-F^ mice (% Area- F(1, 31) = 3.45, p = 0.072; Count- F(1, 31) = 0.08, p = 0.77; ANOVA, Fig 3B and 3C). We then created linear regression models factoring in brain region, perinatal choline diet, and sex to analyze Aβ42-positive percent area, average Aβ42-positive plaque count, and average Aβ42-positive plaque size across the brain in 6-month-old *App*^NL-G-F^ mice. From these models, we found that there was a significant effect of perinatal choline supplementation on Aβ42-positive percent area and Aβ42-positive plaque average size but not Aβ42-positive plaque count (% Area- F(1, 94) = 7.91, p = 0.006; Size- F(1, 94) = 19.25, p<0.0001; Count- F(1, 94) = 0.38, p = 0.54; ANOVA, Fig 3D). This effect was that 6-month-old *App*^NL-G-F^ mice that were fed the choline supplemented diet perinatally showed significantly less Aβ42-positive percent area and had a significantly smaller average Aβ42-positive plaque size compared to 6-month-old *App*^NL-G-F^ mice that were raised on the control diet perinatally (Fig 3D).

In the amygdala of 9-month-old *App*^NL-G-F^ mice, perinatal choline supplementation dramatically reduced Aβ42-positive percent area, average Aβ42-positive plaque count, and average Aβ42-positive plaque size compared to 9-month-old *App*^NL-G-F^ mice that received the control diet (% Area- F(1, 33) = 6.94, p = 0.013; Count- F(1, 33) = 4.87, p = 0.035; Size- F(1, 33) = 7.75, p = 0.009; ANOVA, Fig 3A and 3C). Matching the amygdala, in the cortex of 9-month-old *App*^NL-G-F^ mice, perinatal choline supplementation also significantly reduced Aβ42-positive percent area, average Aβ42-positive plaque count, and average Aβ42-positive plaque size compared to control diet 9-month-old *App*^NL-G-F^ mice (% Area- F(1, 33) = 10.23, p = 0.003; Count- F(1, 33) = 6.07, p = 0.019; Size- F(1, 33) = 9.77, p = 0.004; ANOVA, Fig 3B and 3C). Perinatal choline supplementation significantly reduced Aβ42-positive percent area but not average Aβ42-positive plaque count and average Aβ42-positive plaque size in the hippocampus of 9-month-old *App*^NL-G-F^ mice (% Area- F(1, 33) = 7.49, p = 0.0099; Count- F(1, 33) = 1.61, p = 0.213; Size- F(1, 33) = 1.32, p = 0.259; ANOVA, Fig 3B and 3C). We again created linear regression models factoring in brain region, perinatal choline diet, and sex to analyze Aβ42-positive percent area, average Aβ42-positive plaque count, and average Aβ42-positive plaque size across the brain in 9-month-old *App*^NL-G-F^ mice. Using these linear regression models, we found that perinatal choline supplementation significantly reduced Aβ42-positive percent area, average Aβ42-positive plaque count, and average Aβ42-positive plaque size across the brain in 9-month-old *App*^NL-G-F^ mice (% Area- F(1, 99) = 20.70, p<0.0001; Count- F(1, 99) = 8.33, p = 0.005; Size- F(1, 99) = 16.37, p = 0.0001; ANOVA, Fig 3D).

In the amygdala of 12-month-old *App*^NL-G-F^ mice, perinatal choline supplementation did not significantly reduce Aβ42-positive percent area, average Aβ42-positive plaque count, or average Aβ42-positive plaque size (% Area- F(1, 28) = 1.58, p = 0.22; Count- F(1, 28) = 3.29; p = 0.08; Size- F(1, 28) = 1.50, p = 0.23; ANOVA, Fig 3B and 3C). Perinatal choline supplementation reduced Aβ42-positive percent area but not average Aβ42-positive count or average Aβ42-positive plaque size compared to those that received the control diet perinatally in the cortex of 12-month-old *App*^NL-G-F^ mice (% Area- F(1, 27) = 4.54, p = 0.042; Count- F(1, 27) = 3.13, p = 0.09; Size- F(1, 27) = 2.39, p = 0.13; ANOVA, Fig 3B and 3C). In the hippocampus of 12-month-old *App*^NL-G-F^ mice, there was no effect of perinatal choline supplementation on Aβ42-positive percent area, Aβ42-positive plaque count, or average Aβ42-positive plaque size (% Area- F(1, 27) = 0.99, p = 0.33; Count- F(1, 27) = 0.10, p = 0.76; Size- F(1, 27) = 2.12, p = 0.16; ANOVA, Fig 3B and 3C). When using linear regression models adjusting for brain region, perinatal choline diet, and sex, for the three Aβ42 measures in 12-month-old *App*^NL-G-F^ mice, we found that perinatal choline supplementation significantly reduced Aβ42-positive percent area and Aβ42-positive plaque size but not average Aβ42-positive plaque size (% Area- F(1, 82) = 7.14, p = 0.009; Size- F(1, 82) = 5.84, p = 0.018; Count- F(1, 82) = 0.59, p = 0.44; ANOVA, Fig 3D).

We lastly created linear regression models accounting for brain region, perinatal choline diet, sex, and age to analyze Aβ42-positive percent area, Aβ42-positive plaque count, and average Aβ42-positive plaque size in *App*^NL-G-F^ mice. We found an overall significant effect of age on Aβ42-positive percent area, average Aβ42-positive plaque count, and average Aβ42-positive plaque size (% Area- F(3, 114.5) = 84.91, p<0.0001; Count- F(3, 114.2) = 133.17, p<0.0001; Size- F(3, 112.5) = 14.14, p<0.0001; repeated measures ANOVA, Fig 3D). *Post-hoc* analysis revealed that 6-, 9-, and 12-month-old *App*^NL-G-F^ mice had a higher Aβ42-positive percent area than 3-month-old *App*^NL-G-F^ mice (3-Months (3.15 ± 0.3%); 6-Months (7.13 ± 0.3%); 9-Months (9.68 ± 0.3%); 12-Months (11.25 ± 0.3%); p<0.0001 for all; Tukey, Fig 3D). Nine- and 12-month-old *App*^NL-G-F^ mice had a significantly higher Aβ42-positive percent area than 6-month-old *App*^NL-G-F^ mice (6-Months (7.13 ± 0.3%) vs 9-Months (9.68 ± 0.3%) p<0.0001; 6-Months (7.13 ± 0.3%) vs 12-Months (11.25 ± 0.3%) p<0.0001; Tukey, Fig 3D). Twelve-month-old *App*^NL-G-F^ mice had a significantly higher Aβ42 percent area than 9-month-old *App*^NL-G-F^ mice (9-Months (9.68 ± 0.3%) vs 12-Months (11.25 ± 0.3%) p = 0.0008; Tukey, Fig 3D). Six-, 9-, and 12-month-old *App*^NL-G-F^ mice had an increased average Aβ42-positive plaque count compared to 3-month-old *App*^NL-G-F^ mice (3-Months (31.34 ± 2.1); 6-Months (66.16 ± 1.8); 9-Months (82.62 ± 1.8); 12-Months 98.41 ± 2.0); p<0.0001 for all; Tukey, Fig 3D). Nine- and 12-month-old *App*^NL-G-F^ mice had an increased Aβ42-positive plaque count compared to 6-month-old *App*^NL-G-F^ mice (6-Months (66.16 ± 1.8); 9-Months (82.62 ± 1.8); 12-Months (98.41 ± 2.0); p<0.0001 for both; Tukey, Fig 3D). Twelve-month-old *App*^NL-G-F^ mice had an increased average Aβ42-positive plaque count compared to 9-month-old *App*^NL-G-F^ mice (9-Months (82.62 ± 1.8) vs 12-Months 98.41 ± 2.0) p<0.0001; Tukey, Fig 3D). Six-, 9-, and 12-month-old *App*^NL-G-F^ mice had a significantly larger average Aβ42-positive plaque size than 3-month-old *App*^NL-G-F^ mice (3-Months (40.71 ± 1.2); 6-Months (50.29 ± 0.9); 9-Months (50.12 ± 1.0); 12-Months (52.17 ± 1.1); p<0.0001 for all comparisons, Tukey, Fig 3D). Interestingly, 6-, 9-, and 12-month-old *App*^NL-G-F^ mice showed no difference in their average Aβ42-positive plaque size, suggesting that average Aβ42-positive plaque size is increased up until 6-months of age where it levels off (6-Months (50.29 ± 1.0) vs 9-Months (50.12 ± 1.0) p = 0.999; 6-Months (50.29 ± 1.0) vs 12-Months (52.17 ± 1.1) p = 0.557; 9-Months (50.12 ± 1.0) vs 12-Months (52.17 ± 1.1) p = 0.476; Tukey, Fig 3D). Importantly, we found that perinatal choline supplementation reduced Aβ42-positive percent area by around 18%, reduced average Aβ42-positive plaque count by more than 5%, and reduced average Aβ42-positive plaque size by more than 10% in *App*^NL-G-F^ mice across the brain across all ages (% Area- Control (8.51 ± 0.3%) vs Supplemented (7.07 ± 0.2%) F(1, 114.6) = 15.75, p<0.0001; Count- Control (71.50 ± 1.7) vs Supplemented (67.51 ± 1.6) F(1, 114.3) = 4.05, p = 0.047; Size- Control (50.91 ± 0.9) vs Supplemented (45.64 ± 0.9) F(1, 112.7) = 17.08, p<0.0001; repeated measures ANOVA, Fig 3E). These results provide further evidence that perinatal choline supplementation is able to alter the deposition of Aβ42 in the brains of AD model mice and that Aβ42 deposition occurs in an age-dependent manner in *App*^NL-G-F^ mice.

## Discussion

We investigated the effects of gestational and early postnatal choline supplementation on the cognitive behavioral and brain amyloid deposition in the *App*^NL-G-F^ AD mouse model. Consistent with previous choline supplementation studies in other AD mouse models, perinatal choline supplementation in *App*^NL-G-F^ mice improved both spatial and fearful learning and memory at various ages and significantly reduced amyloidosis in the amygdala, cortex, and hippocampus.

We first tested our animals in the Open Field Test and Elevated Plus Maze to assess gross locomotor function and anxiety-related behavior. Importantly, as previously reported, there were no differences in locomotor activity between wildtype mice and *App*^NL-G-F^ mice [27, 29, 31, 32]. In line with previous studies, we observed subtle exploratory and anxiety-related differences in the Open Field and Elevated Plus Maze at later ages (9- and 12-months-old) between wildtype and *App*^NL-G-F^ mice, [27, 31, 32]. Overall, these differences are unlikely to influence measures of learning and memory utilized in this study.

Most commonly, the first symptoms AD patients report are issues with learning and memory (Reviewed in [39]). In early stages of the disease, working memory as well as longer-term declarative memory are impacted and drastically affect a patient’s quality of life and day-to-day activities. Spatial learning and memory rapidly decline in AD patients much faster than in normal aging (Reviewed in [40]). Spatial learning and memory are largely dependent on the hippocampus-entorhinal cortex circuit which is greatly affected by the pathology of AD. We assessed hippocampal-dependent spatial learning and memory using the Barnes Maze [36] and found a significant deficit of spatial learning in *App*^NL-G-F^ mice as compared to wildtype mice during the trial phase of the maze at 3-, 6-, and 12-months of age. These deficits were abolished by perinatal choline supplementation at early ages (3 and 6 months) but not at the later age (12 months) suggesting that choline supplementation is able to prevent early spatial learning deficits that are found in *App*^NL-G-F^ mice. Previous results of *App*^NL-G-F^ mice in the Barnes Maze have varied [24, 29, 30]. One study that used male and female 6–9-month-old mice, also found a significant increase in latency to reach the target hole in *App*^NL-G-F^ mice compared to wildtype mice [29]. Another study that used 6-month-old mice with 9 training days for their trial phase found no difference in latency to reach the target hole between wildtype and *App*^NL-G-F^ mice [30] and another study found only deficits in *App*^NL-G-F^ mice as compared to wildtype mice, in the trial phase of the Barnes Maze at 8 months of age but not at 4 or 6 months of age [24]. In these studies, only male mice were used while in our study both male and female mice were used. Interestingly, we do see deficits in *App*^NL-G-F^ males, as compared to wildtype males, at 3-, 6-, and 12-months of age. Additionally, Sakakibara and colleagues were testing the animals in the Barnes Maze longitudinally at 4-, 6-, and 8-months-old [24] while our study utilized a cross-sectional design where the animals were only tested at one age enabling us to not have any interference from memories of previous testing. Furthermore, our sample size for each timepoint was greater than previous studies as previous studies ranged from having 6–10 animals [24] and 23–25 [30] per genotype per age while in our study each genotype ranged from 32–39 animals per genotype at each age. Having more animals per group, we have better statistical power to detect a change in behavior. Our study animals were also housed in a reverse light cycle and tested during their more active light-off period, while some other studies conducted their testing during the light-on period. This difference in timing of testing conditions could alter how the mice perform and be leading to some of the discrepancies with previous studies. We also used purified diets for both the control and choline supplemented mice while others have used normal mouse chow. Our control diet contains about 53% less choline than the diet used in Hongo et al.’s study [30] and may also contribute to the slight differences we see in Barnes Maze performance. Interestingly, when using the Morris Water Maze, Mehla and colleagues [23] found that *App*^NL-G-F^ mice had impaired hippocampal-dependent spatial learning as early as 6-months of age and continued up until 12-months of age. Together, our results further support the notion that *App*^NL-G-F^ mice have impaired spatial learning. We report that this phenotype is modifiable as perinatal choline supplementation prevents these hippocampal-dependent learning deficits at early ages in *App*^NL-G-F^ mice. The reduced penetrance of the *App*^NL-G-F^ mutations on these behavioral phenotypes by high perinatal choline intake may also explain some of the above-described discrepancies in the literature as the nutritional status of the mice used in these studies was not controlled.

AD patients also suffer from non-declarative associative learning and memory deficits (Reviewed in [41]). To study this, we utilized a contextual fear conditioning paradigm and found that 3- and 6-month-old *App*^NL-G-F^ mice had no impairments in this test as compared to wildtype mice. However, we found impairments in associating the tone with the footshock in both 9- and 12-month-old *App*^NL-G-F^ mice. Remarkably, perinatal choline supplementation prevented this fearful learning and memory deficit in 9-month-old and 12-month-old *App*^NL-G-F^ mice. Additionally, both 9- and 12-month-old *App*^NL-G-F^ mice showed significant impairment in remembering the context in which the fear-inducing footshocks occurred compared to wildtype mice. Perinatal choline supplementation prevented these deficits in *App*^NL-G-F^ mice at both 9- and 12-months of age. Our findings are consistent with the results of Kundu and colleagues [28] who reported a significant impairment in *App*^NL-G-F^ mice during the tone test of 6-month-old mice. One other study using only male 6-9-month-old mice, also found no difference in freezing behavior during the context test [24]. This same study found no significant difference in freezing during the context test between *App*^NL-G-F^ and wildtype mice that were 15-18-months-old, whereas we observed that 9- and 12-month-old *App*^NL-G-F^ mice had significant fearful memory impairments. However, our contextual fear conditioning paradigm significantly differed from the one of Sakakibara and colleagues [24] as no tone was associated with the footshocks in their study. They were interested in the animal’s behavior to associate the footshock with the environment/context it occurred in while our study was designed to evaluate the association of both a tone and the context in which the fear-inducing footshocks occurred. This can partially explain why these results differ. While no previous studies have investigated the effects of choline supplementation in AD mouse models on contextual fear conditioning, previous studies investigating choline supplementation on developmental alcohol and nicotine exposure have found that choline supplementation can prevent fearful learning and memory deficits involved with these conditions [42–44]. Our results further support the notion that choline supplementation is able to prevent fearful learning and memory deficits.

Our data confirm previous reports that amyloid-beta deposition occurs in an age-dependent manner in *App*^NL-G-F^ mice [22, 23]. We now report that perinatal choline supplementation can markedly reduce Aβ42 deposition in the cortex, amygdala, and hippocampus of *App*^NL-G-F^ mice. This finding is consistent with previous studies which found that choline supplementation—whether perinatally or lifelong—decrease amyloid deposition in other AD mouse models [10, 16, 17, 38]. We have previously shown that in the APP.PS1 mouse model of AD, perinatal choline supplementation significantly increases β-CTF protein levels in the hippocampus of 12-month-old mice, reminiscent of what is seen in other AD models when treated with a γ-secretase inhibitor [10]. Thus, the reason for reduced Aβ in choline supplemented animals may be through the inhibition or reduction of γ-secretase activity. Interestingly, Velazquez and colleagues found that, in their lifelong choline supplemented APP.PS1 mice, there was a significant decrease in β-CTF protein levels [16]. They propose that this finding, coupled with reduced brain levels of soluble and insoluble levels of Aβ40 and Aβ42, indicate a reduction in the amyloidogenic processing of the APP protein and lead to the reduction of Aβ burden in choline supplemented animals. They further suggest and show that Aβ burden reduction via choline supplementation is accompanied by a reduction in the brain levels of the neurotoxic amino acid homocysteine [38]. It is known that homocysteine binds and facilitates aggregation and accumulation of Aβ and therefore it is plausible that life-long choline supplementation is reducing Aβ through this mechanism [16, 45]. Furthermore, high levels of homocysteine are a known risk factor for the development of AD in humans and targeting homocysteine for the treatment of AD has been proposed [45–47]. There is evidence that high choline intake is associated with low homocysteine levels in humans [48, 49]. However, this mechanism is unlikely to be relevant for the current study which employed choline supplementation in early life, as the homocysteine-level-lowering action of choline is mediated by betaine—the product of enzymatic oxidation of choline—and thus requires contemporaneous supply of choline.

We have previously reported that perinatal choline supplementation increases mRNA and protein levels of insulin-like growth factor 2 (IGF2) [50, 51]. Additionally, infusion of IGF2 in the APP.PS1 mouse model of AD ameliorated amyloidosis [52]. Another study by Xia et al. found that IGF2 mRNA was markedly reduced in AD patients and when they treated Tg2576 AD model mice with IGF2 they improved memory consolidation, decreased Aβ deposition and reduced oxidative stress through the activation of the PI3K/AKT pathway [53].

There are numerous reports that show choline supplementation is capable of reducing oxidative stress in a variety of animal models [54–58]. Oxidative stress and mitochondria dysfunction are characteristically found in AD patients and restoration of mitochondrial function is currently being targeted as a strategy to delay or slow the progression of AD (Reviewed in [59]). Oxidative stress is also accompanied with activation of the immune system (Reviewed in [60]). The brains of AD patients are characterized by chronic neuroinflammation and chronic neuroinflammation is associated with synapse damage, synapse loss, and functional brain network dysfunction [61–63]. Furthermore, *App*^NL-G-F^ mice display neuronal and synaptic deficits along with marked neuroinflammation [23, 64–66]. Perinatal choline supplementation prevented astrogliosis in APP.PS1 mice [10] and life-long choline supplementation decreased the expression of activated microglial markers [16]. Microglia are phagocytic immune cells of the central nervous system and are critical for response to damage in the brain. Microglial activation state and phenotype is dependent on a complex environment in which they reside and can lead to varying states of efficiency for handling Aβ and other damage. It is thought that microglia in AD transition to a phenotype that is less efficient at phagocytosing Aβ plaques and lead to further accumulation of Aβ and synaptic deficits [61, 67]. Targeting of neuroinflammation, and microglia specifically, is being investigated pre-clinically and in clincial trials for AD and choline supplementation may be a viable way to modulate neuroinflammatory states in AD. Together, this suggests that perinatal choline supplementation may be leading to decreased Aβ42 deposition and improved cognitive performance through the reduction of amyloidogenic APP processing, oxidative stress and mitochondrial dysfunction that then leads to a dampened immune response and synaptic deficits in *App*^NL-G-F^ mice.

Choline may also modulate DNA methylation through its conversion into betaine, leading to the production of S-adenosylmethionine that is eventually used to methylate DNA. Indeed, maternal choline supplementation leads to an increase in overall S-adenosylmethionine levels, a decrease in 5-methylcytosine content in genomic DNA, and a reduction in DNA methyltransferase *Dnmt1* and *Dnmt3a* mRNA levels in the brain of fetal rats [68]. Gestational choline supply also regulates methylation of histones [1, 69, 70]. Methylation of DNA and histones are mechanisms used to regulate gene expression and since choline availability is capable of affecting both of these processes, it may modulate the expression of genes that are important for learning and memory, brain cell proliferation, migration, and apoptosis during development, that lead to lifelong changes [4, 7, 71–74]. Indeed, we have previously shown that maternal choline supply in rats increases the expression of genes whose protein products participate in the processes of learning and memory, including calcium/calmodulin-dependent protein kinase I (*Camk1*) and *Igf2* in the cortex and of the transcription factor *Zif268/Egr1* in the cortex and hippocampus [10]. Future studies are needed to investigate the effects of perinatal choline supplementation in wildtype and *App*^NL-G-F^ mice on DNA methylation and mRNA expression and to assess if they correlate with the improved performance of the *App*^NL-G-F^ mice in learning and memory tasks.

Although our study provides a comprehensive evaluation of the behavioral and Aβ pathological characterization of the *App*^NL-G-F^ AD mouse model and the effects of perinatal choline supplementation within the model on these measures across the lifespan, this study has limitations as it did not examine mechanisms of these effects. Future work in areas such as brain network connectivity, gene and/or protein expression, or DNA methylation that were outside the scope of this study will be needed to address this problem. Another limitation is due to the cross-sectional nature of our study that did not allow for the animals to complete behavioral testing at various time points across the lifespan within the same animal. With that being said, using a cross-sectional design did prevent repeated testing and confounding bias caused by repeated testing in our animals.

In conclusion, our study shows that the amyloidosis and AD-related cognitive deficits that characterize the *App*^NL-G-F^ mice are markedly slowed or prevented, respectively, by a nutritional strategy with high maternal choline intake during pregnancy and lactation. There are implications of these observations to human health, namely that adequate choline intake by pregnant and nursing women may similarly protect their children from age-related dementias and AD. The current estimates of the consumption of choline indicate that only approximately 8% of pregnant women in the United States meet the recommended adequate intake (AI) values [75]. Similar results have been reported for pregnant women in Australia [76]. Our current data, as well as previous studies cited above, support the notion that formulation and implementation of public health policies designed to ensure that choline intake be consistent with the AI values would be a valuable tool for the prevention of many cases of dementia and AD.

## Supporting information

S1 Table Animal counts used for behavioral testing. (DOCX)

S2 Table Animal counts used for Aβ42 immunohistochemistry. (DOCX)

S1 Fig Experimental design. Mating pairs were placed on experimental diets 7 days before mating commenced. Mothers were kept on experimental diets until time of weaning of pups at post-natal day 21. All experimental mice received the control diet after weaning. Mice were tested behaviorally in the Open Field, Elevated Plus Maze, Barnes Maze, and Contextual Fear Conditioning paradigm starting around 3-, 6-, 9-, or 12-months of age and euthanized one week after completion of behavior testing.
(DOCX)

S2 Fig Areas of interest for amygdala, cortex, and hippocampus used for Aβ42 immunohistocehmistry quantification. Consistent regions of interest that encompassed the basolateral and basomedial amygdalar nuclei, the primary somatosensory cortex, and the hippocampus were used for Aβ42 quantification and shown below in the 2x magnification images.
(DOCX)

S3 Fig *App*^NL-G-F^ mice display subtle anxiety-related differences in the open field test at 9- and 12-months of age. (A) Total distance traveled throughout the entire Open Field Test revealed no differences in locomotor activity between wildtype and *App*^NL-G-F^ mice at any age. There were also no differences in distance traveled due to perinatal choline supplementation. (B) *App*^NL-G-F^ spent significantly more time in the center of the Open Field Test than wildtype mice at 9- and 12-months of age (F(1, 65) = 5.5014, p = 0.0221 & F(1, 62) = 5.5143, p = 0.0221, respectively, ANOVA). Nine-month-old wildtype mice that received the control diet spent significantly more time in the center than 9-month-old *App*^NL-G-F^ mice that also received the control diet (Control WT (163.52 ± 13.4s) vs Control *App*^NL-G-F^ (124.65 ± 12.9s) p = 0.017; Tukey). No differences were found between wildtype and *App*^NL-G-F^ mice at 3- or 6-months of age.
(DOCX)

S4 Fig *App*^NL-G-F^ mice display altered anxiety-related behaviors in the elevated plus maze. (A) *App*^NL-G-F^ mice did not differ from wildtype mice in time spent in the open arms or middle of the Elevated Plus Maze at 3- (F(1, 64) = 0.2344, p = 0.627), 6- (F(1, 67) = 2.9829, p = 0.089), or 9-months of age (F(1, 65) = 0.5874, p = 0.446; ANOVA). *App*^NL-G-F^ mice spent significantly more time in the open arms or middle than wildtype mice at 12-months of age (F(1, 62) = 6.9130, p = 0.0108, ANOVA, black bars). Mice that received the choline supplemented diet spent significantly more time in the open arms or middle at 3-months of age (F(1, 64) = 4.5354, p = 0.037; ANOVA) but less time at 12-months of age than those that received the control diet perinatally (F(1, 62) = 4.6142, p = 0.036; ANOVA, blue bars). (B) No differences between wildtype and *App*^NL-G-F^ mice were found in percentage of open arm entries when combining diet groups and when looking within both control and choline supplemented groups. There was a significant overall effect of diet in 3-month-old mice as those that received the choline supplemented diet made a significantly higher percentage of open arm entries than mice the received the control diet (F(1, 64) = 5.45, p = 0.023; ANOVA, blue bars). No other diet differences were detected.
(DOCX)

S5 Fig Female mice performed significantly better than male mice in the Barnes maze. (A) During the trial day period, there was a significant effect of sex as female mice found the target hole significantly faster than male mice at 3-, 6-, 9-, and 12-months of age (3-Months- F(1, 64) = 6.07, p = 0.016; 6-Months- F(1, 67) = 5.00, p = 0.029; 9-Months- F(1, 64) = 15.45, p = 0.0002; 12-Months- F(1, 62) = 7.78, p = 0.007; repeated measures ANOVA). (B) There was also a significant effect of sex in the 1-day probe test as female mice found the target hole significantly faster than male mice at 6-, 9-, and 12-months of age (6-Months- F(1, 67) = 7.92, p = 0.006; 9-Months- F(1, 65) = 15.89, p = 0.0002; 12-Months- F(1, 62) = 6.86, p = 0.011; ANOVA).
(DOCX)

S6 Fig Subtle sex differences in contextual fear conditioning performance. (A) Female mice froze significantly less during the tone test than male mice at 3-months of age (F(1, 64) = 4.87, p = 0.0309; ANOVA). No sex differences were found at 6-, 9-, or 12-months of age in the tone test. (B) There was a significant effect of sex in 12-month-old mice during the context test as male mice froze significantly more than female mice (F(1, 62) = 7.15, p = 0.0096; ANOVA). No other sex differences were found at any other age in the context test.
(DOCX)

S1 File Supporting text for the open field test and elevated plus maze. (DOCX)
