# Supplementary material for: Perinatal choline supplementation prevents learning and memory deficits and reduces brain amyloid Aβ42 deposition in AppNL-G-F Alzheimer’s disease model mice
Source: PLoS One. 2024 Feb 5;19(2):e0297289. doi: 10.1371/journal.pone.0297289 (PMC10843108; doi:10.1371/journal.pone.0297289)
Supplement: S1 Table — (DOCX) [file pone.0297289.s001.docx]

| **Animals Used for Behavioral Testing** | | | | | | |
| --- | --- | --- | --- | --- | --- | --- |
| **Perinatal Diet** | **Genotype** | **Sex** | **3 Months** | **6 Months** | **9 Months** | **12 Months** |
| Control Diet | Wildtype | Male | 9 | 8 | 7 | 10 |
|  |  | Female | 11 | 13 | 9 | 10 |
|  | *App*^NL-G-F^ | Male | 7 | 8 | 9 | 9 |
|  |  | Female | 8 | 8 | 8 | 5 |
| Choline Supplemented Diet | Widltype | Male | 11 | 11 | 9 | 10 |
|  |  | Female | 7 | 7 | 8 | 8 |
|  | *App*^NL-G-F^ | Male | 10 | 10 | 11 | 9 |
|  |  | Female | 9 | 10 | 10 | 9 |
