# Supplementary figures and images for: Perinatal choline supplementation prevents learning and memory deficits and reduces brain amyloid Aβ42 deposition in AppNL-G-F Alzheimer’s disease model mice
Source: PLoS One. 2024 Feb 5;19(2):e0297289. doi: 10.1371/journal.pone.0297289 (PMC10843108; doi:10.1371/journal.pone.0297289)

**
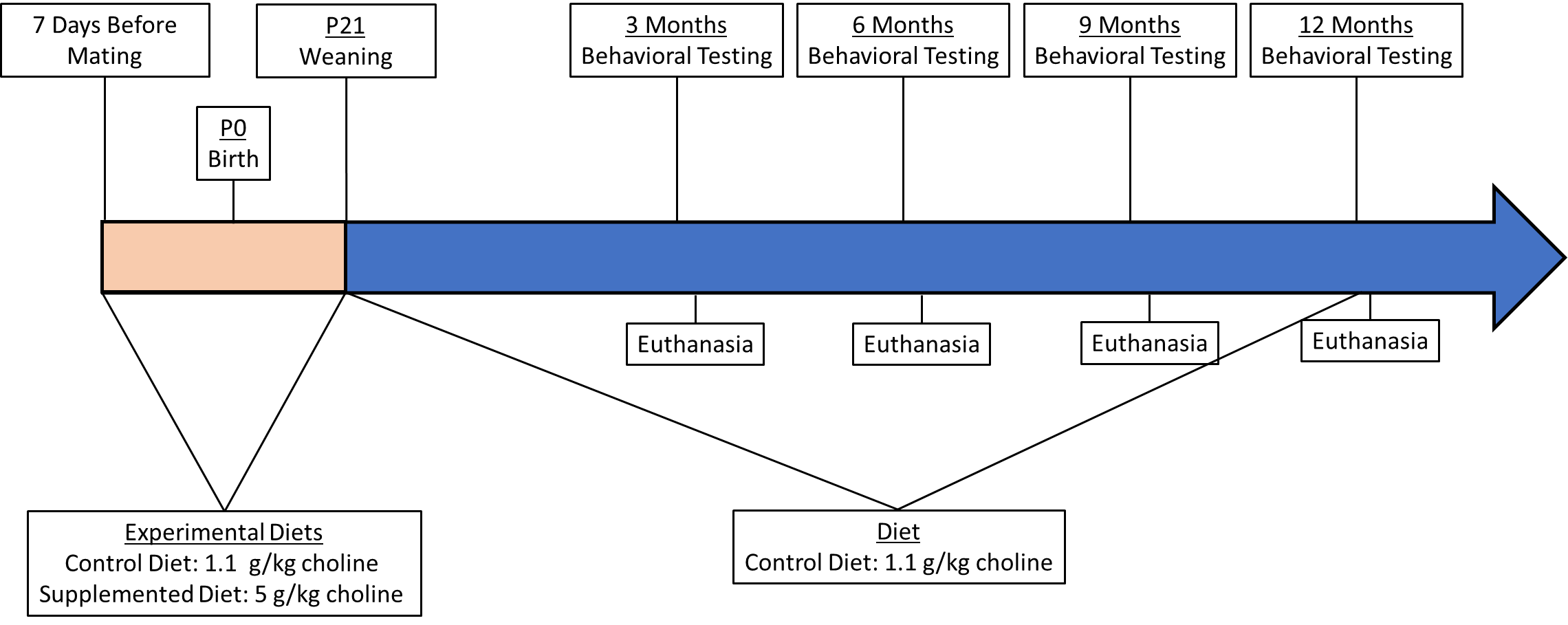
**

Supplement: S1 Fig — Mating pairs were placed on experimental diets 7 days before mating commenced. Mothers were kept on experimental diets until time of weaning of pups at post-natal day 21. All experimental mice received the control diet after weaning. Mice were tested behaviorally in the Open Field, Elevated Plus Maze, Barnes Maze, and Contextual Fear Conditioning paradigm starting around 3-, 6-, 9-, or 12-months of age and euthanized one week after completion of behavior testing. (DOCX) [file pone.0297289.s003.docx]

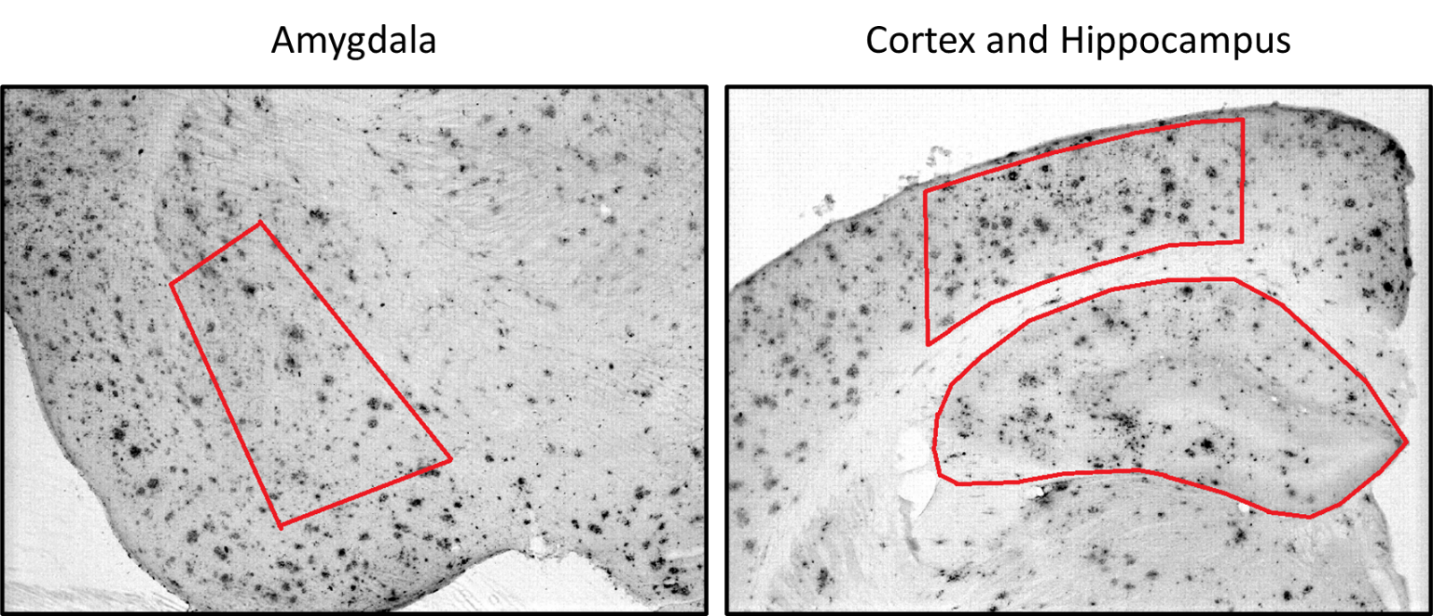

Supplement: S2 Fig — Consistent regions of interest that encompassed the basolateral and basomedial amygdalar nuclei, the primary somatosensory cortex, and the hippocampus were used for Aβ42 quantification and shown below in the 2x magnification images. (DOCX) [file pone.0297289.s004.docx]

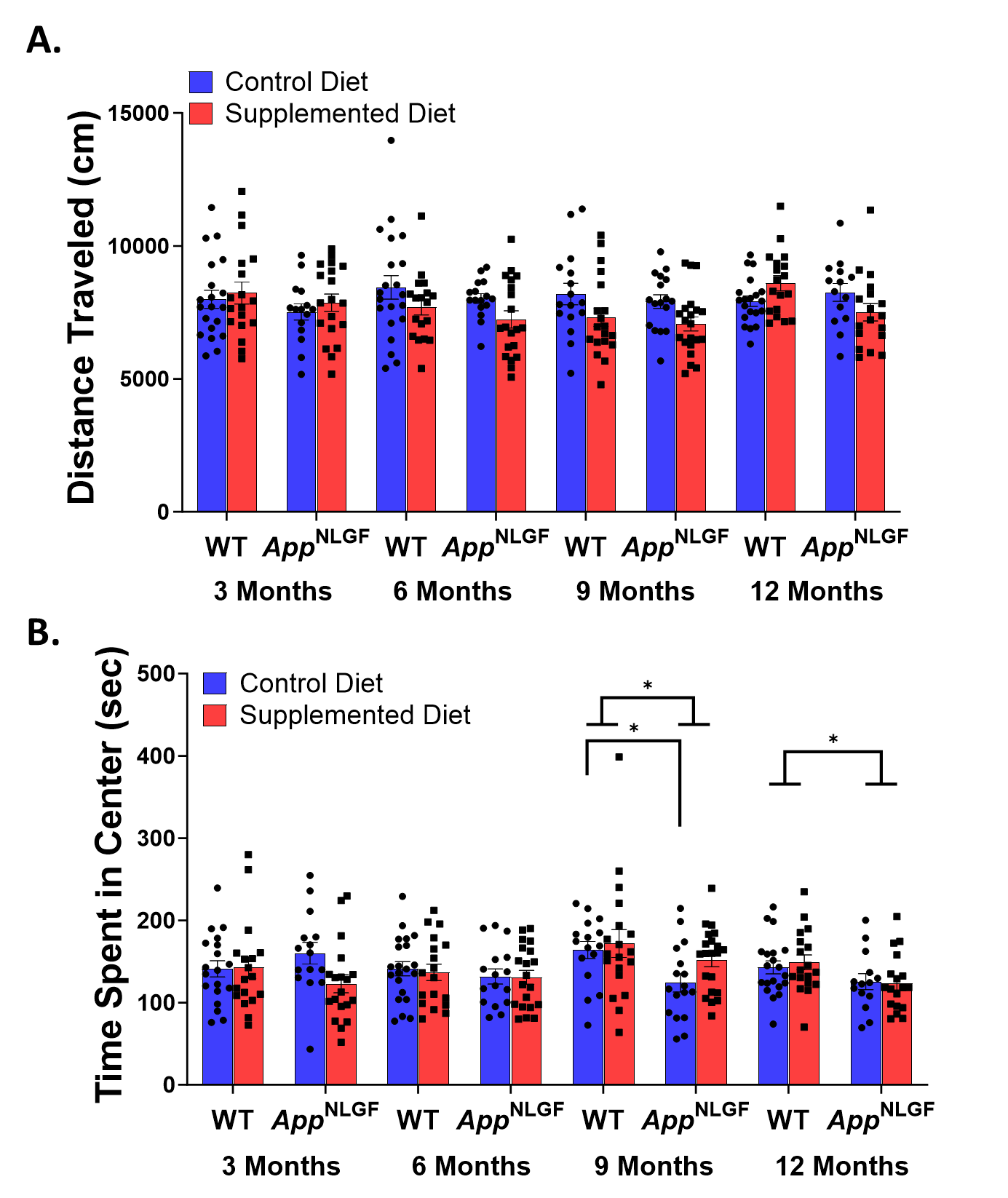

Supplement: S3 Fig — (A) Total distance traveled throughout the entire Open Field Test revealed no differences in locomotor activity between wildtype and AppNL-G-F mice at any age. There were also no differences in distance traveled due to perinatal choline supplementation. (B) AppNL-G-F spent significantly more time in the center of the Open Field Test than wildtype mice at 9- and 12-months of age (F(1, 65) = 5.5014, p = 0.0221 & F(1, 62) = 5.5143, p = 0.0221, respectively, ANOVA). Nine-month-old wildtype mice that received the control diet spent significantly more time in the center than 9-month-old AppNL-G-F mice that also received the control diet (Control WT (163.52 ± 13.4s) vs Control AppNL-G-F (124.65 ± 12.9s) p = 0.017; Tukey). No differences were found between wildtype and AppNL-G-F mice at 3- or 6-months of age. (DOCX) [file pone.0297289.s005.docx]

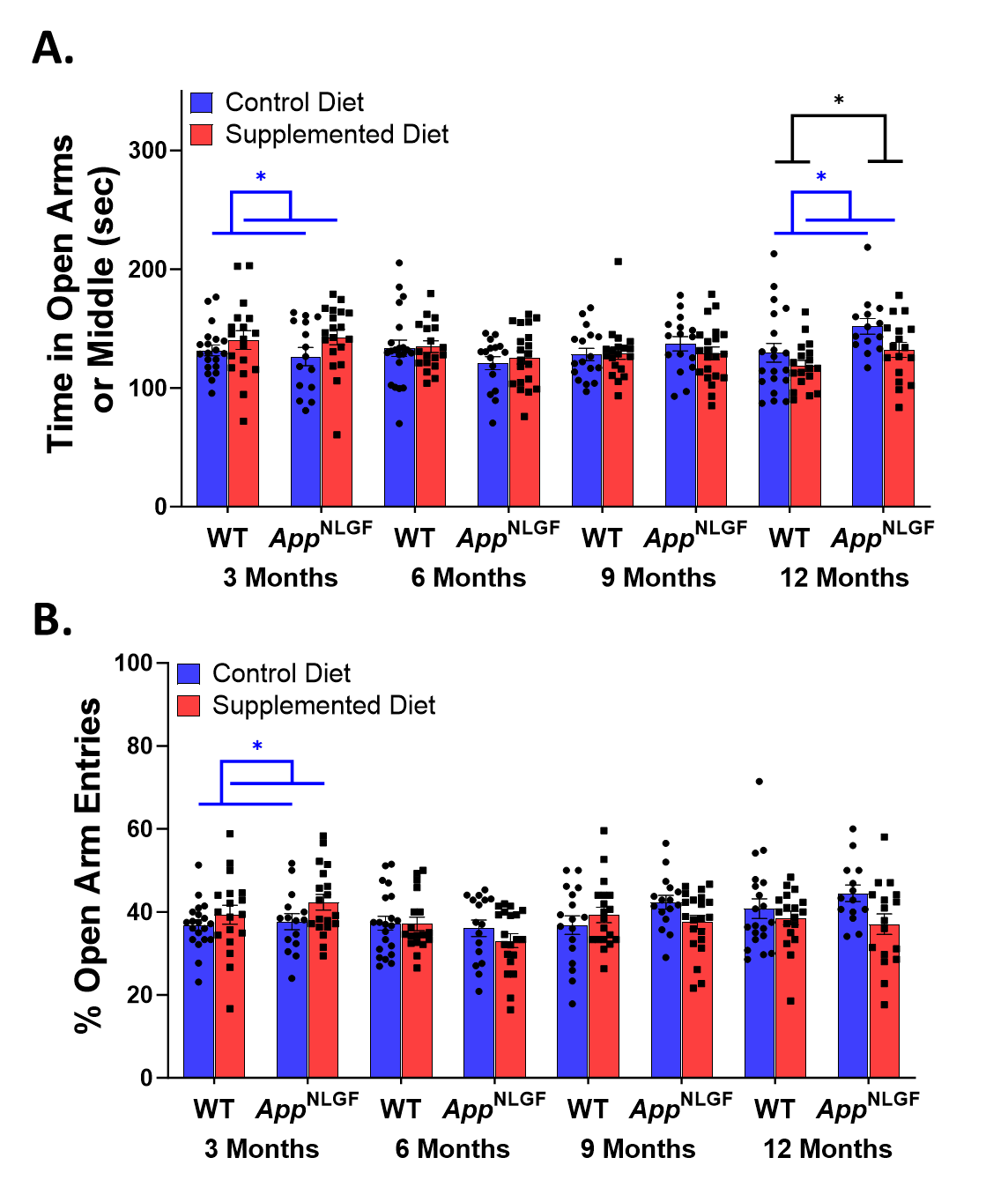

Supplement: S4 Fig — (A) AppNL-G-F mice did not differ from wildtype mice in time spent in the open arms or middle of the Elevated Plus Maze at 3- (F(1, 64) = 0.2344, p = 0.627), 6- (F(1, 67) = 2.9829, p = 0.089), or 9-months of age (F(1, 65) = 0.5874, p = 0.446; ANOVA). AppNL-G-F mice spent significantly more time in the open arms or middle than wildtype mice at 12-months of age (F(1, 62) = 6.9130, p = 0.0108, ANOVA, black bars). Mice that received the choline supplemented diet spent significantly more time in the open arms or middle at 3-months of age (F(1, 64) = 4.5354, p = 0.037; ANOVA) but less time at 12-months of age than those that received the control diet perinatally (F(1, 62) = 4.6142, p = 0.036; ANOVA, blue bars). (B) No differences between wildtype and AppNL-G-F mice were found in percentage of open arm entries when combining diet groups and when looking within both control and choline supplemented groups. There was a significant overall effect of diet in 3-month-old mice as those that received the choline supplemented diet made a significantly higher percentage of open arm entries than mice the received the control diet (F(1, 64) = 5.45, p = 0.023; ANOVA, blue bars). No other diet differences were detected. (DOCX) [file pone.0297289.s006.docx]

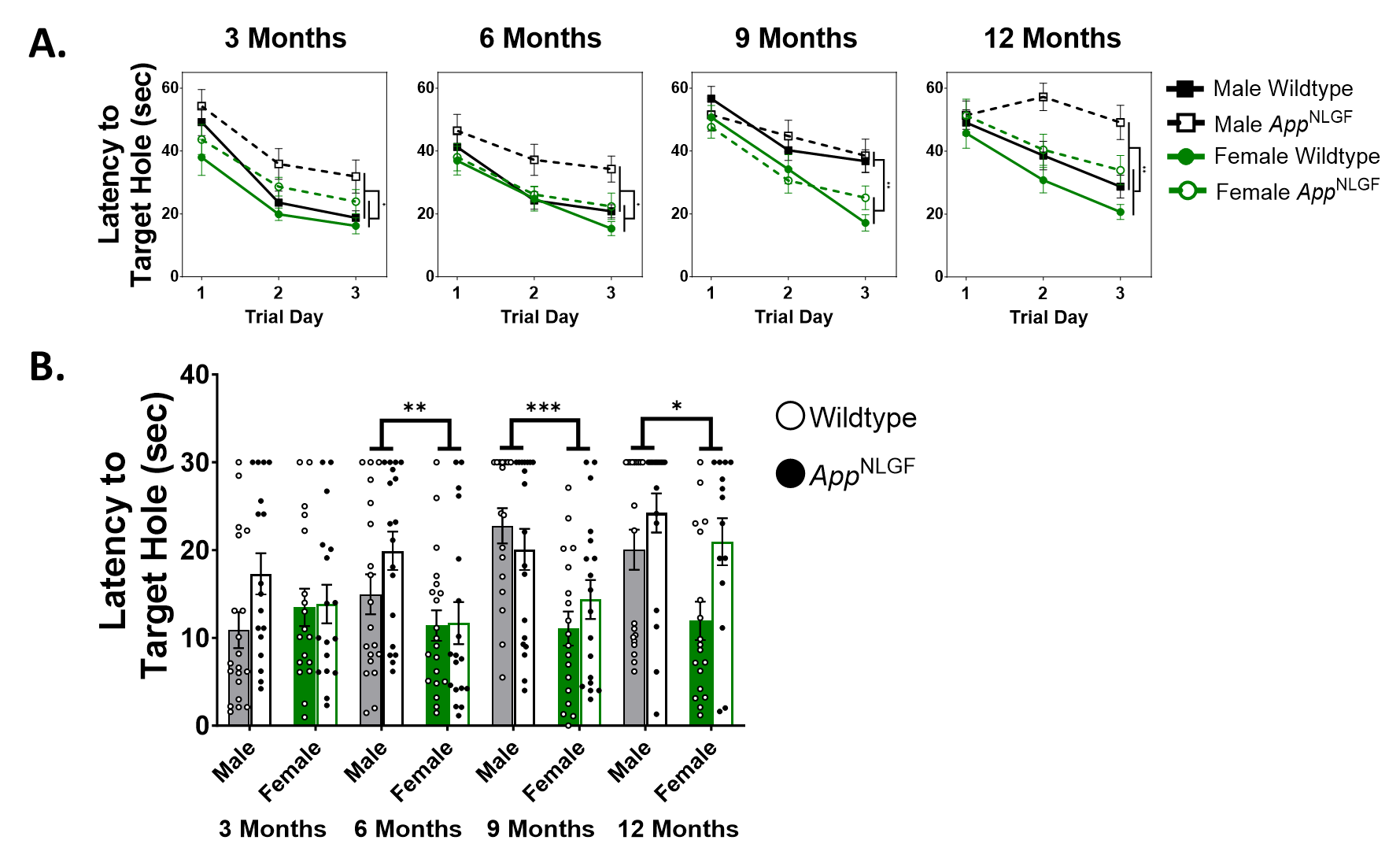

Supplement: S5 Fig — (A) During the trial day period, there was a significant effect of sex as female mice found the target hole significantly faster than male mice at 3-, 6-, 9-, and 12-months of age (3-Months- F(1, 64) = 6.07, p = 0.016; 6-Months- F(1, 67) = 5.00, p = 0.029; 9-Months- F(1, 64) = 15.45, p = 0.0002; 12-Months- F(1, 62) = 7.78, p = 0.007; repeated measures ANOVA). (B) There was also a significant effect of sex in the 1-day probe test as female mice found the target hole significantly faster than male mice at 6-, 9-, and 12-months of age (6-Months- F(1, 67) = 7.92, p = 0.006; 9-Months- F(1, 65) = 15.89, p = 0.0002; 12-Months- F(1, 62) = 6.86, p = 0.011; ANOVA). (DOCX) [file pone.0297289.s007.docx]

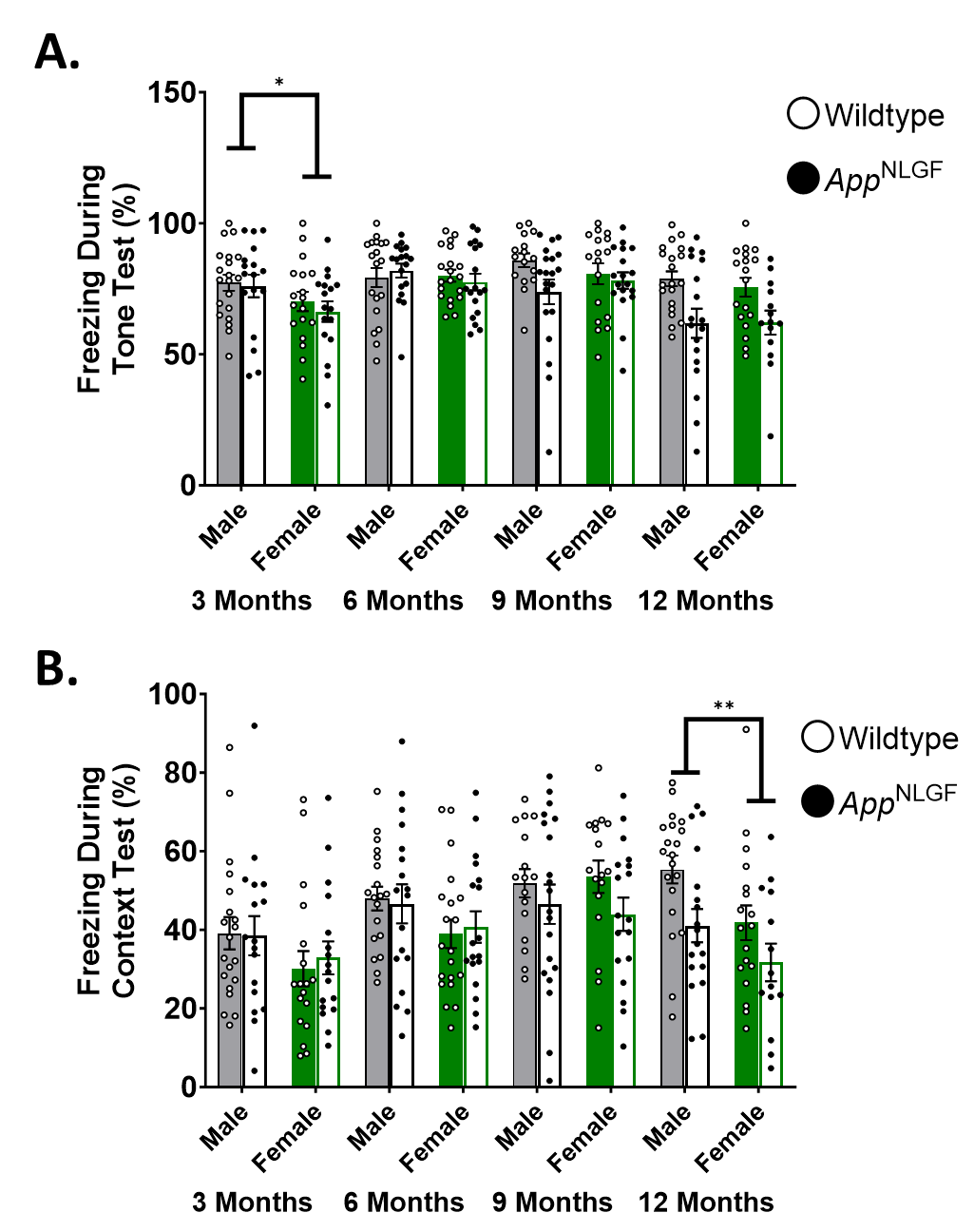

Supplement: S6 Fig — (A) Female mice froze significantly less during the tone test than male mice at 3-months of age (F(1, 64) = 4.87, p = 0.0309; ANOVA). No sex differences were found at 6-, 9-, or 12-months of age in the tone test. (B) There was a significant effect of sex in 12-month-old mice during the context test as male mice froze significantly more than female mice (F(1, 62) = 7.15, p = 0.0096; ANOVA). No other sex differences were found at any other age in the context test. (DOCX) [file pone.0297289.s008.docx]
