# Supplementary material for: Perinatal choline supplementation prevents learning and memory deficits and reduces brain amyloid Aβ42 deposition in AppNL-G-F Alzheimer’s disease model mice
Source: PLoS One. 2024 Feb 5;19(2):e0297289. doi: 10.1371/journal.pone.0297289 (PMC10843108; doi:10.1371/journal.pone.0297289)
Supplement: S1 File — (DOCX) [file pone.0297289.s009.docx]

**Supporting text for Open Field and Elevated Plus Maze**

*Open Field Test Results*

We first explored whether *App*^NL-G-F^ mice displayed any locomotor or anxiety-related differences compared to the wildtype mice using the Open Field Test. When broken down into 5-minute intervals, we found that all groups (wildtype, *App*^NL-G-F^, male, female, control diet, choline supplemented diet, and all combinations of these) steadily decreased total distance traveled and increased the amount of time spent in the center of the arena over time during the 15-minute trial at 3-, 6-, 9, and 12-months of age as expected (p<0.05, repeated measures ANOVA). At 3 and 6 months of age, there were no overall differences due to genotype in overall distance traveled (3-Months: F(1, 64)= 1.28, p=0.262; 6-Months: F(1, 67)= 1.27, p=0.263; ANOVA, Fig S3A) or total time spent in center of the arena (3-Months: F(1, 64)= 0.0002, p=0.990; 6-Months: F(1, 67)= 0.43, p=0.518; ANOVA, S3A & S3B Fig) between wildtype and *App*^NL-G-F^ mice. There was an overall effect of genotype in 9-month-old mice on time spent in the center as 9-month-old *App*^NL-G-F^ mice spent significantly less time in the center of the Open field than their 9-month-old wildtype counterparts (F(1, 65)= 5.50, p=0.022; ANOVA, S3B Fig). Interestingly, *post-hoc* analysis revealed that the genotype difference on total time spent in the center of the Open Field was only apparent in control *App*^NL-G-F^ mice (124.6 ± 12.9s) compared to control wildtype mice (163.5 ± 13.4s) (p=0.049, Tukey, S3B Fig). There was also a significant effect of genotype in 12-month-old mice as *App*^NL-G-F^ mice spent significantly less time in the center of the Open Field Test than 12-month-old wildtype mice (F(1, 62)= 5.51, p= 0.022, ANOVA, S3B Fig). However, this genotype effect was not seen within either individual control diet or choline supplemented diet groups individually (Control WT (142.7 ± 8.2s) vs Control *App*^NL-G-F^ (124.9 ± 10.2s) p=0.524; Supplemented WT (147.4 ± 8.6s) vs Supplemented *App*^NL-G-F^ (123.3 ± 8.6s) p= 0.209; Tukey, S3B Fig). There were no overall diet differences between on total time spent in the center at any of the time points (S3B Fig). There were also no effects of genotype or perinatal diet on total distance traveled during the Open Field Test at any of the ages tested (S3A Fig). These results indicate that there is a very subtle effect of the *App*^NL-G-F^ genotype on exploration in a novel environment only at 9- and 12-months of age and that there is no locomotor dysfunction in *App*^NL-G-F^ mice.

*Elevated Plus Maze Results*

We further investigated behavioral and anxiety-related behaviors using the Elevated Plus Maze. We found that there was no significant effect of genotype on both time spent in the open arms or in the middle and percentage of open arm entries at 3-months of age (Time in Open Arms or Middle: F(1, 64)= 0.23, p=0.630; Percent of Open Arm Entries: F(1, 64)= 0.52, p=0.475; ANOVA, S4A & S4B Fig). Interestingly, we found that there was an overall effect of diet as 3-month-old mice that received the choline supplemented diet spent significantly more time in the open arms or the middle and had a higher percentage of open arm entries than 3-month-old mice that received the control diet (Time in Open Arms or Middle: F(1, 64)= 4.54, p=0.037; Percent of Open Arm Entries: F(1, 64)=5.45, p=0.023; ANOVA, S4A & S4B Fig). At 6- and 9-months of age, there were no differences between wildtype and *App*^NL-G-F^ mice in both time spent in the open arms or the middle of the Elevated Plus Maze or in percentage of open arm entries in diet separated groups or in diet combined groups (Supplemental Figure 4A & 4B). There was also no significant effect of diet on time spent in the open arms or middle or in percentage of open arm entries in 6- or 9-month-old animals (Supplemental Figure 4A & 4B). In 12-month-old mice, there was a significant effect of genotype as 12-month-old *App*^NL-G-F^ mice spent significantly more time in the open arms or in the middle of the Elevated Plus Maze than 12-month-old wildtype mice (F(1, 62)= 6.91, p=0.011; ANOVA, S4A Fig). There was also a significant effect of perinatal choline supplementation as 12-month-old mice that received the choline supplemented diet spent significantly more time in the open arms or middle of the Elevated Plus Maze than 12-month-old mice that received the control diet (F(1, 62)= 4.61, p=0.036; ANOVA, S4A Fig). There were no genotype or perinatal diet differences on percentage of open arm entries in 12-month-old mice (Supplemental Figure 4B). These results suggest that there are minimal effects of both the *App*^NL-G-F^ genotype and perinatal choline supplementation on anxiety-related behavior in the Elevated Plus Maze.
